# Experimental and computational insights into the therapeutic mechanisms of resveratrol in a *Drosophila* α-synuclein model of Parkinson’s disease

**DOI:** 10.1038/s41598-025-00698-9

**Published:** 2025-05-22

**Authors:** Amos Olalekan Abolaji, Adeola Oluwatosin Adedara, Judith Chizoba Madu, Oluwabunmi Tomilola Owalude, Oludare Michael Ogunyemi, Damilola A. Omoboyowa, Folorunsho Bright Omage, Alexander J. Whitworth, Michael Aschner

**Affiliations:** 1 Drosophila Laboratory, Molecular Drug Metabolism and Toxicology Unit, Department of Biochemistry, Faculty of Basic Medical Sciences, College of Medicine, University of Ibadan, Ibadan, Oyo State Nigeria; 2 Drosophila Research and Training Centre, Basorun, Ibadan, Nigeria; 3 Structural and Computational Biology Group, Nutritional and Industrial Biochemistry Research Unit, Department of Biochemistry, Faculty of Basic Medical Sciences, College of Medicine, University of Ibadan, Ibadan, Oyo State Nigeria; 4 Department of Biochemistry, Adekunle Ajasin University, Akungba-Akoko, Ondo State Nigeria; 5 Programa de Pós-Graduação em Ciências Biológicas: Bioquímica Toxicológica, Universidade Federal de Santa Maria, Santa Maria, Brazil; 6 MRC Mitochondrial Biology Unit, University of Cambridge, Cambridge Biomedical Campus, Hills Road, Cambridge, CB2 0XY UK; 7 Department of Molecular Pharmacology, Albert Einstein College of Medicine, New York, USA

**Keywords:** α-synuclein, Antioxidant, Parkinson’s disease, Resveratrol, Network pharmacology, Molecular docking, Cellular neuroscience, Diseases of the nervous system, Biochemistry, Neuroscience, Parkinson's disease

## Abstract

Parkinson’s disease (PD) is a multifactorial neurodegenerative disorder driven by genetic predisposition and environmental exposure. Given its well-documented antioxidative and neuroprotective properties, resveratrol is increasingly being considered for its potential to counteract the neuronal damage characteristic of Parkinson’s disease. Here, we investigated the therapeutic action of resveratrol in a transgenic *Drosophila melanogaster* model expressing human α-synuclein (*SNCA*, PD flies), in combination with network pharmacology and molecular docking analyses. The PD flies were fed diet supplemented with resveratrol (15, 30, and 60 mg/kg diet, approximately 6.57, 13.14 and 26.28 mM, respectively), to evaluate lifespan. This was followed by a 21-day treatment of PD flies with similar concentrations of resveratrol in the diet to evaluate cognitive function, oxidative stress, and antioxidant biomarkers, using Levodopa (0.1 mM) as positive control. The results showed that resveratrol supplementation in the diet significantly improved lifespan, locomotor activity, acetylcholinesterase and catalase activities, and thiol content compared to untreated PD flies. Furthermore, resveratrol reduced nitric oxide (nitrite/nitrate), malondialdehyde, and total hydroperoxide levels, and enhanced cellular metabolic activity and upregulated *Sod1* mRNA expression (*p* < 0.05). The network pharmacology and molecular docking analyses identified key molecular targets that may account for the therapeutic action of resveratrol, including B-Cell Lymphoma 2, Monoamine Oxidase (MAO); in flies, MAO-Like, Dopa Decarboxylase, Protein Kinase A and Glycogen Synthase Kinase-3 (GSK-3). Among these, MAO and GSK-3 emerged as top targets as indicated by network prominence and strong binding interactions. Additionally, the binding interaction of resveratrol to SNCA at specific sites suggests a potential role in inhibiting its aggregation, which is a hallmark of PD pathology. Quantum mechanics calculations revealed that resveratrol functions as both a proton donor and acceptor, contributing to its strong target binding interactions and antioxidant potential. Overall, resveratrol supplementation in the diet may be beneficial for PD management by modulating dopamine metabolism, apoptosis, oxidative stress, and cell survival. The study provides valuable experimental and computational insights into the underlying therapeutic mechanisms of action of resveratrol and supports its potential use in PD management.

## Introduction

Parkinson’s disease (PD) ranks as the second most prevalent neurodegenerative disorder globally and a leading cause of motor dysfunction. It is pathologically depicted by dopaminergic neuronal loss in the substantia nigra pars compacta in association with α-synuclein (SNCA) aggregation^1^. The underlying biological mechanisms of PD are yet to be fully elucidated. However, key contributing factors include abnormal protein aggregation, mitochondrial impairment, oxidative stress and neuroinflammatory processes^2^. The SNCA is a neuronal protein localised in the presynaptic terminals in healthy conditions. In a soluble monomeric form, SNCA regulates the release of neurotransmitters, vesicle exocytosis, and homeostasis^3^. Mutations and dysregulation in the *SNCA*, which encodes the α-synuclein protein, have been linked to the development of both inherited and sporadic cases of PD^4^. Mutations in *SNCA* lead to the formation of SNCA oligomers, which aggregate into Lewy bodies in the brain^3^ to cause a dominant form of parkinsonism. The accumulation of these Lewy body aggregates plays an important role in the pathology of PD, as SNCA aggregates affect many cellular mechanisms, thereby causing cytotoxicity^5^ and ultimately cell death^6^. The role of SNCA in PD development and its connection to the normal physiological function of α-synuclein remains poorly understood. Inflammation, mitochondrial dysfunction and oxidative stress are three interconnected mechanisms that contribute to SNCA-mediated PD^7^. Notably, elevated expression of SNCA has been shown to impair mitochondrial function, triggering oxidative stress and promoting apoptotic cell death^8^.

Excessive production of either normal or mutated SNCA in *Drosophila melanogaster* neurons affords optimal models for the study of PD^9,10^. Research has shown that Heat Shock Protein 70 (HSP70) could inhibit the adverse effects of SNCA in yeast and fly models of PD^11^. Furthermore, the introduction of diverse expression-control siRNAs in transgenic fly models of PD resulted in suppression of *SNCA* to varying degrees, which was found to be associated with enhancements in motor performance^12^. Gispert et al.^13^ demonstrated that *PINK1* could inhibit symptoms of SNCA, including poor movement, cell degeneration, and reduced longevity. The administration of Geldanamycin, a compound that enhances the activity of the HSP 70 chaperone, has been shown to mitigate dopaminergic neurodegeneration and synuclein aggregation in flies with *SNCA* mutations^14^.

Due to the lack of effective PD therapy and management, strategies to identify therapies that can cross the blood–brain barrier are imperative. Dopamine replacement therapy has been used since the 1960s as a treatment for PD^15^ as it compensates for the loss of dopamine. Drugs such as the dopamine precursor Levodopa (L-DOPA), dopamine agonists, and monoamine oxidase B inhibitors are commonly used. The effects of L-DOPA, however, wear off as the disease progresses and often result in adverse side effects, including dyskinesias and motor fluctuations^16^. Although numerous neuroprotective compounds have been evaluated in various PD models, many have proven ineffective due to poor blood–brain barrier permeability or limited bioavailability.

Neuropathological studies in early-stage PD have revealed increased oxidative stress markers, suggesting that oxidative damage precedes significant neuronal loss. Oxidative stress results when there is an imbalance between Reactive Oxygen Species (ROS) generation and the body’s antioxidant defense system^17^. Since oxidative stress is a multi-targeted pathological process, agents mitigating oxidative stress could be tailored to the management of neurodegenerative diseases, including PD. Growing interest in natural polyphenols has been driven by their potential neuroprotective benefits and their emerging role in combating oxidative damage in these conditions^18^. The functional hydroxyl group in phenolic compounds mediates their antioxidative property by scavenging free radicals or their ability to chelate metal ions^19^. Various natural polyphenols, such as curcumin, resveratrol, quercetin, genistein, and catechins, have been reported to have protective roles against oxidative stress, neuroinflammation, and mitochondrial dysfunction in neurodegenerative diseases^20^.

Network pharmacology is an emerging discipline that examines the interactions between drugs and biological networks, offering a systems-level understanding of drug actions. Traditional pharmacology often focuses on single-target drug approaches. However, diseases like Parkinson’s, which are characterized by complex pathophysiological mechanisms involving oxidative stress, neuroinflammation, protein misfolding (SNCA aggregation), mitochondrial dysfunction, and dopaminergic neuronal loss^21,22^, require a multi-target approach. In network pharmacology, the nodes and edges form the framework of a biological network, where the connections between nodes represent the biochemical or pharmacological interactions that underlie health and disease states. This enables identification of the hub genes with high connectivity and central roles in maintaining the network integrity. These hub genes are often key regulators of biological processes and may serve as crucial therapeutic targets. This aligns well with the goals of personalized medicine, where understanding the individual variability in a patient’s biological network can help tailor drug therapies. For example, in Parkinson’s disease, different patients may have different underlying causes (e.g., varying degrees of mitochondrial dysfunction or oxidative stress). A number of studies have highlighted the identification of phytochemicals with multi-target potential against parkinsonism using network pharmacological approaches^23–25^. While network pharmacology offers valuable insights into potential therapeutic targets, combining it with molecular docking and other computational modeling techniques provides a deeper understanding of how these compounds interact with various targets^26–28^. This integrative strategy allows for the modulation of multiple nodes within biological networks, as shown in several studies, thereby advancing the discovery of multi-target agents and improving therapeutic strategies for complex disorders like neurodegenerative diseases^29,30^.

Resveratrol (trans-3,5,6-trihydroxystilbene) is a popular nonflavonoid polyphenol dietary supplement naturally found in grapes and berries; and commonly consumed in red wine^31^. It has been reported to have chemoprotective^32^, anti-inflammatory, anti-oxidative^33,34^, and neuroprotective properties^35^. Recently, the lifespan-extending property^36^ and rescue action of resveratrol in *parkin* loss-of-function-induced oxidative stress was reported^37^. The therapeutic potential of resveratrol in Parkinson’s disease has been extensively reported in various experimental models^38–40^. However, the mechanism underpinning this activity is still unfolding. Apart from this, resveratrol has been used in different clinical trials for different ailments. For instance, a 1.0 g dose of resveratrol administered twice daily for 52 weeks increased adaptive immunity in Alzheimer’s disease subjects^41^. Additionally, administration of 400 mg per day dose of resveratrol improved endothelial function in subjects with chronic kidney diseases and diabetes^42^.

Given the significant similarity in genetic and molecular biology between flies and humans and the proven utility of using *Drosophila* as a simple animal model for PD, we sought to experimentally explore the therapeutic action of resveratrol in a fly model of PD transgenically expressing human *SNCA* in combination with in silico analysis of human targets.

## Results

### Resveratrol extends the lifespan of PD *Drosophila melanogaster*

**Fig. 1:**
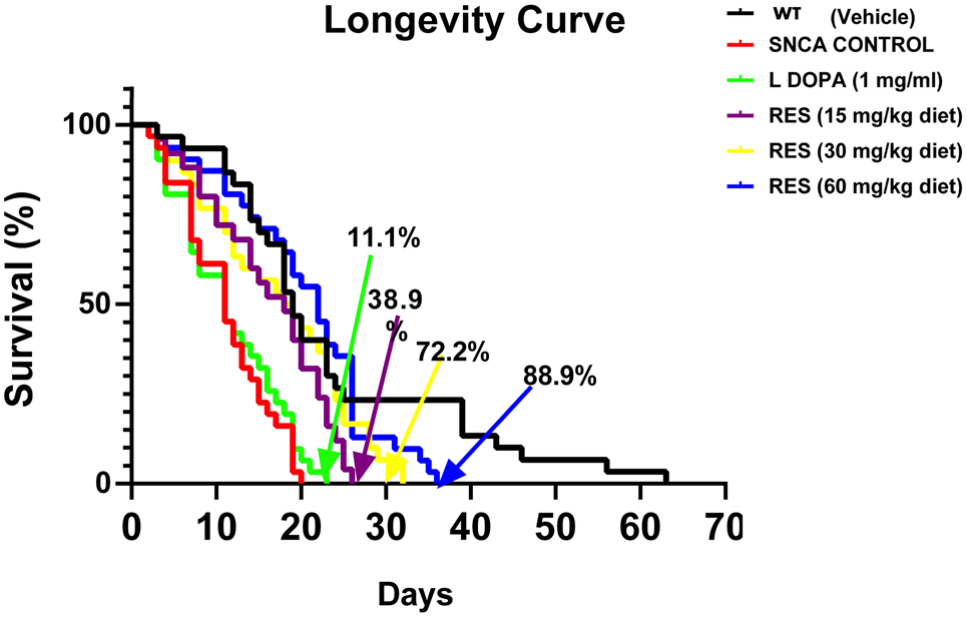
Action of resveratrol on lifespan of PD *D. melanogaster*. The maximum lifespan in each group represents the percentage of surviving flies.

The *SNCA* PD fly model is characterised by substantial motor deficits and shortened lifespan^9,10^. Firstly, lifespan analysis was carried out in control (*w*^*1118*^, (vehicle alone, 2% ethanol final concentration in diet)), and PD flies (neuronally overexpressing *SNCA*) treated with L-DOPA (200 µL of 1.0 mg/mL of L-DOPA mixed with 10 g diet (0.1 mM)) and resveratrol (0, 15, 30 and 60 mg/kg diet), respectively (Fig. 1). The results showed that PD flies had a 65% decrease in maximal lifespan when compared to the control. The PD flies exposed to 15 mg/kg diet (approximately 6.57 mM), 30 mg/kg diet (approximately, 13.14 mM) and 60 mg/kg diet (approximately, 26.28 mM) of resveratrol improved lifespan by 38.9, 72.2, and 88.9%, respectively, when compared with the PD flies exposed to vehicle alone. By comparison, L-DOPA, a common drug used in the management of PD, improved the survival of PD flies by 11.1% compared with untreated PD flies.

### Resveratrol improves locomotor performance and Acetylcholinesterase activity in PD *Drosophila melanogaster*

**Fig. 2:**
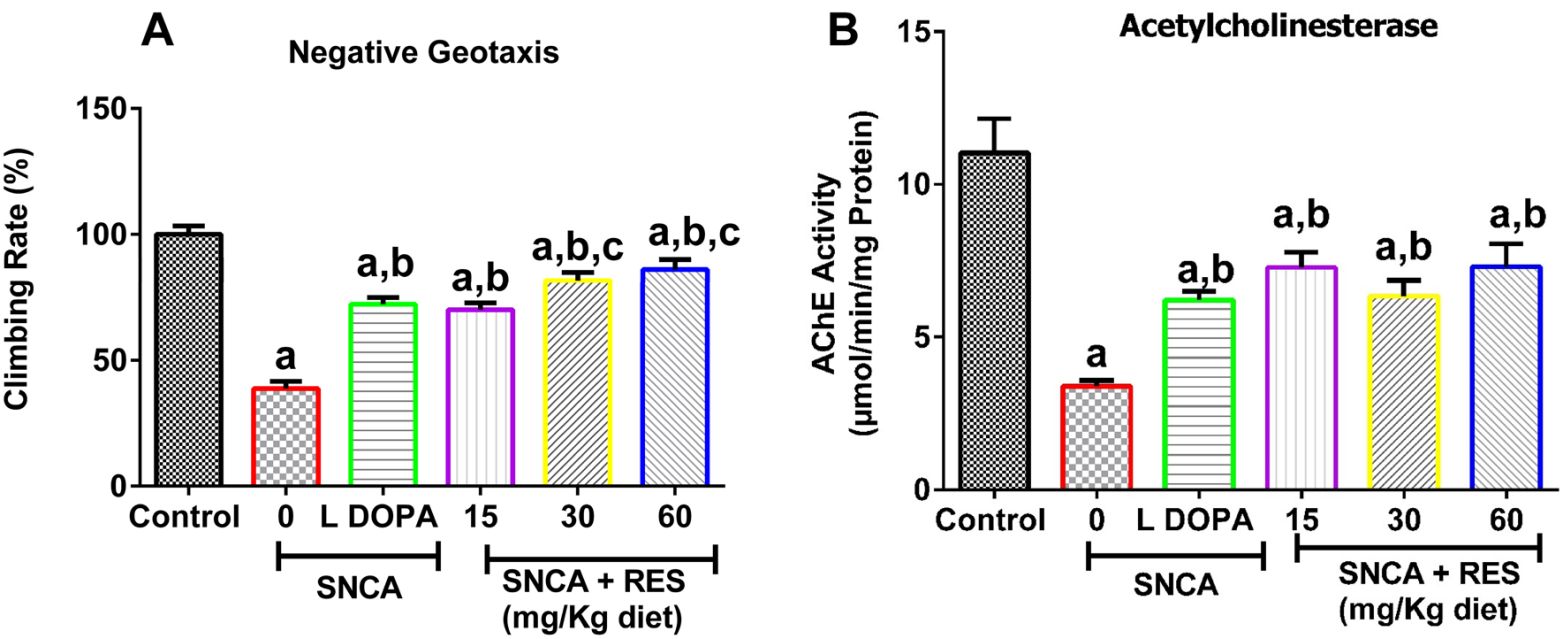
Effects of resveratrol on SNCA model of Parkinson’s disease. Charts are represented as (**A**) Negative Geotaxis and (**B**) Acetylcholinesterase Activity of SNCA model of PD after exposure to resveratrol for 21 days. Values are expressed as Mean ± Standard Error of Mean of 30 flies/vial with 5 replicates per treatment group. Significant differences from the control are indicated by ^**a**^(*p* < 0.05), SNCA PD group are indicated by ^**b**^(*p* < 0.05) and L-DOPA are indicated by ^**c**^(*p* < 0.05).

The SNCA PD flies exhibited a significant reduction in climbing rate by 61% when compared with *w*^*1118*^ control (*p* < 0.05). The PD flies exposed to 15, 30, and 60 mg/kg resveratrol had a dose-dependent increase in locomotor ability by 45, 53, and 55%, respectively (Fig. 2A). Notably, there was significant increase in climbing rate of PD flies fed with L-DOPA and resveratrol when compared with PD flies fed with vehicle alone. Since acetylcholinesterase (AChE) activity has been shown to provide insights into the mechanisms underlying fly’s behaviour, next, we evaluated its activity^43^.We found a significant reduction in AChE activity in PD flies fed with vehicle when compared with *w*^*1118*^ control flies (Fig. 2B). The alteration in the enzyme activity was restored to levels comparable with the control after exposure to resveratrol (*p* < 0.05; Fig. 2B).

### Resveratrol improves antioxidant and redox status in PD *Drosophila melanogaster*

**Fig. 3:**
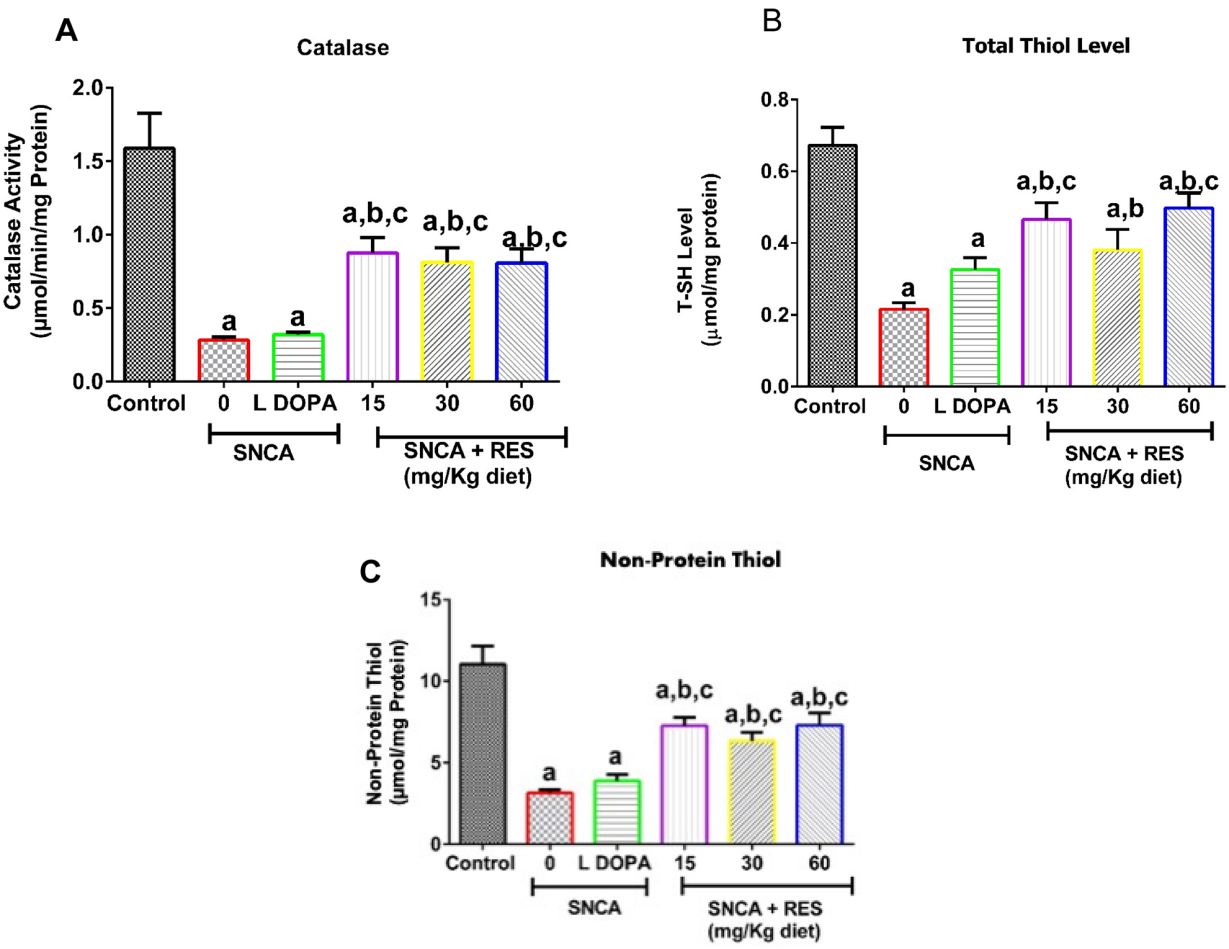
Action of resveratrol on catalase activity, total thiol and non-protein thiol levels of SNCA model of Parkinson’s disease in *D. melanogaster*. Charts are represented as (**A**) Catalase activity (**B**) Total thiol level, and (**C**) non-protein thiol level of PD *D. melanogaster* after exposure to resveratrol for 21 days. Values are expressed as Mean ± Standard Error of Mean of 30 flies/vial with 5 replicates per treatment group. Significant differences from the control are indicated by ^**a**^(*p* < 0.05), SNCA PD group are indicated by ^**b**^(*p* < 0.05) and SNCA PD flies treated with L-DOPA group ^**c**^(*p* < 0.05).

Due to the vital roles play by catalase and thiols in cellular protective mechanisms, we evaluated the activity of catalase as well as total and non-protein thiols in PD flies with and without treatment with resveratrol (Fig. 3). Resveratrol (15, 30 and 60 mg/kg diet) significantly increased catalase activity (Fig. 3A) as well as total thiols and non-protein thiols contents compared to the PD flies fed with the vehicle (Fig. 3B,C, p < 0.05). L-DOPA did not significantly alter catalase activity as well as total and non-protein thiols contents compared with untreated PD flies (*p* > 0.05).

### Resveratrol attenuated PD-induced accumulation of oxidative stress markers in *Drosophila melanogaster*

**Fig. 4:**
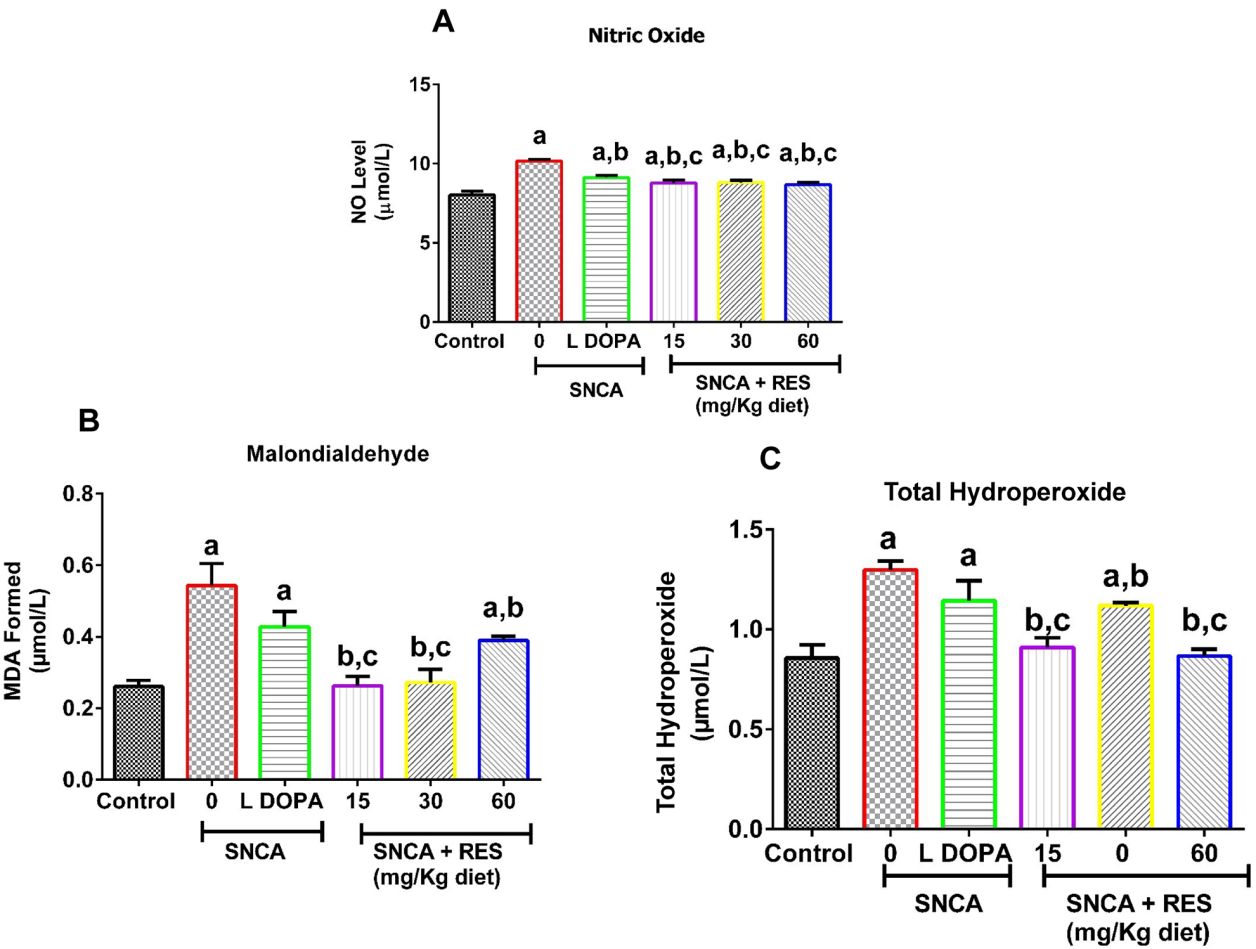
Action of resveratrol on Nitric Oxide level, Lipid Peroxidative Status (measured as Malondialdehyde) and Total Hydroperoxide level of SNCA model of Parkinson’s disease in *D. melanogaster.* Charts are represented as (**A**) Nitric Oxide (**B**) Lipid Peroxidation and (**C**) Total Hydroperoxide level of PD *D. melanogaster* after exposure to resveratrol for 21 days. Values are expressed as Mean ± Standard Error of Mean of 30 flies/vial with 5 replicates per treatment group. Significant differences from the control are indicated by ^**a**^(*p* < 0.05), SNCA PD group are indicated by ^**b**^(*p* < 0.05) and SNCA PD flies treated with L-DOPA group ^**c**^(*p* < 0.05).

The PD flies showed a significant increase in nitric oxide (NO) as indicated by the nitrates/nitrites level (Fig. 4A), malondialdehyde (Fig. 4B), and total hydroperoxide (Fig. 4C), compared with control (*p* < 0.05). The treatment of PD flies with resveratrol significantly attenuated elevations of NO, malondialdehyde and total hydroperoxide levels. In addition, L-DOPA ameliorated PD-induced accumulation of NO, with no effect on hydroperoxide and malondialdehyde levels when compared to PD flies.

### Resveratrol improves cellular metabolic activity of PD *Drosophila melanogaster*

**Fig. 5:**
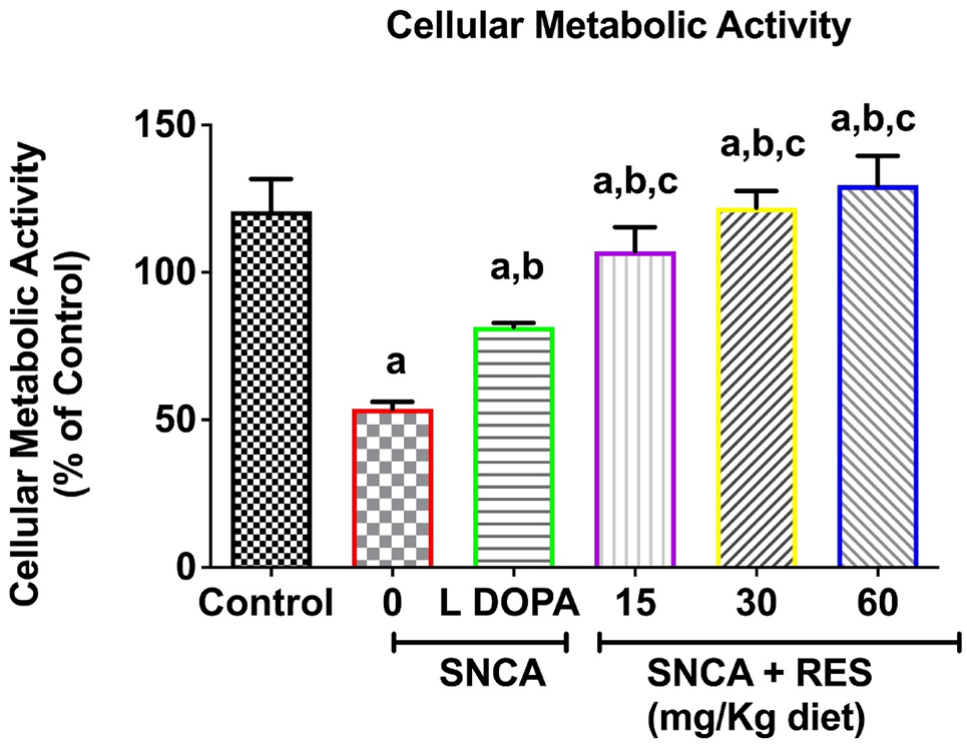
Action of resveratrol on Mitochondrial metabolic rate of SNCA model of Parkinson’s disease in *D. melanogaster.* Charts are represented as cellular metabolic rate of PD *D. melanogaster* after exposure to resveratrol for 21 days. Values are expressed as Mean ± Standard Error of Mean of 30 flies/vial with 5 replicates per treatment group. Significant differences from the control are indicated by ^**a**^(*p* < 0.05), SNCA PD group are indicated by ^**b**^(*p* < 0.05) and SNCA PD flies treated with L-DOPA group ^**c**^(*p* < 0.05).

There was significant reduction in cellular metabolic rate in PD flies compared with control (*p* < 0.05, Fig. 5). A dose-dependent increase in cellular metabolic rate was observed in PD flies treated with resveratrol (15, 30 and 60 mg/kg diets) when compared to untreated PD flies. Additionally, L-DOPA significantly improved the cellular metabolic rate in PD flies compared with untreated PD flies (Fig. 5).

### Resveratrol upregulates *Sod1* gene in PD *Drosophila melanogaster*

**Fig. 6:**
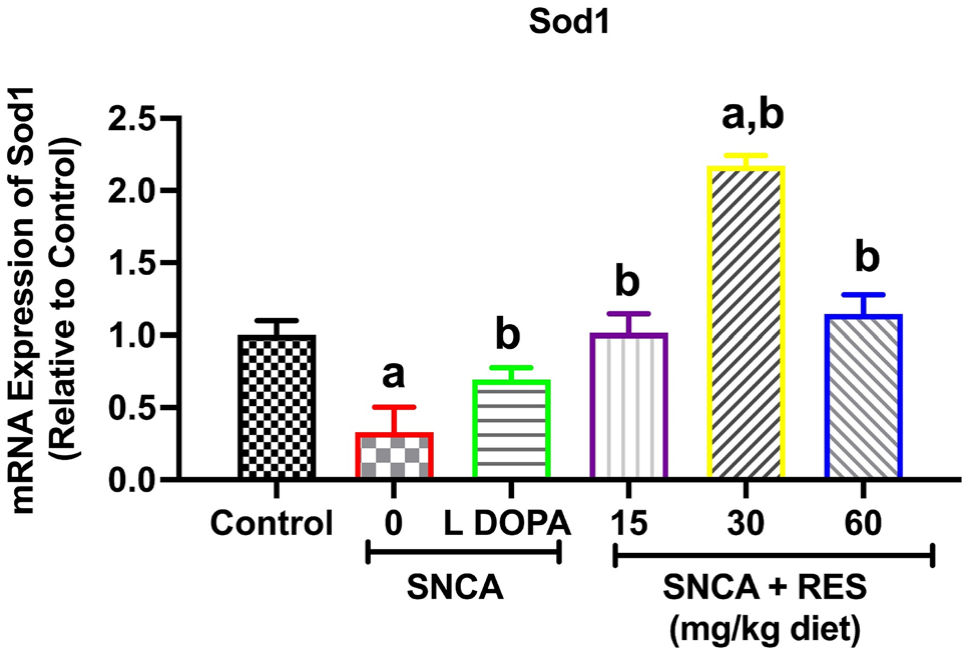
Action of resveratrol on mRNA expression of *Sod1* gene of SNCA model of Parkinson’s disease in *D. melanogaster.* Values are expressed as Mean ± Standard Error of Mean of 30 flies/vial with 5 replicates per treatment group. Significant differences from the control are indicated by ^**a**^(*p* < 0.05), SNCA PD group are indicated by ^**b**^(*p* < 0.05) and SNCA PD flies treated with L-DOPA group ^**c**^(*p* < 0.05).

The expression of *sod1* was examined in this study to understand the mechanism of resveratrol at the molecular level in the SNCA model of PD. The *sod 1* encodes Superoxide Dismutase 1 (Sod 1)^37^, an enzyme responsible for the breakdown of superoxide radicals to molecular oxygen and hydrogen peroxide. Figure 6 shows the mRNA expression level of *sod 1*. A significant reduction was observed in mRNA expression of *sod 1* gene in PD files when compared with *w*^*1118*^ control. However, treatment with resveratrol (15, 30 and 60 mg/kg diet) and L-DOPA increased *sod 1* mRNA level compared with untreated PD flies.

### Prediction of target genes and protein–protein interaction network

**Fig. 7:**
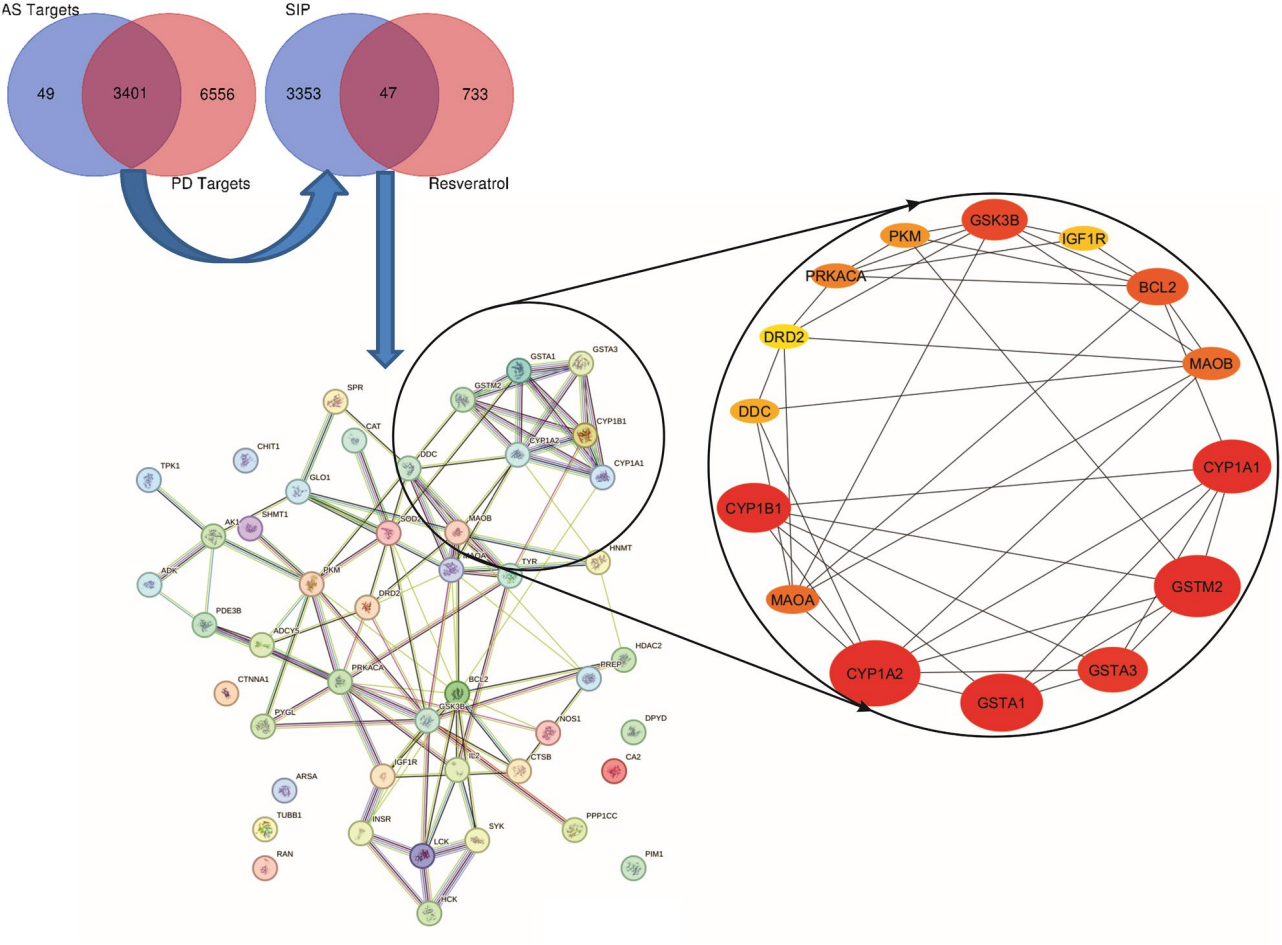
Network Analysis of Resveratrol-Targeted Hub Genes in Parkinson’s Disease (PD) Pathology**.** The Venn diagrams illustrate the overlap between targets of resveratrol, PD-associated genes, and Alzheimer’s Disease (AD)-specific targets. The overlapping region shows 47 common targets between resveratrol and PD, indicating potential therapeutic relevance in PD. The central protein–protein interaction network depicts interactions among these hub genes, with a focus on the highly interconnected proteins related to neurodegenerative pathways. The enlarged network highlights key hub genes involved in PD and their associations. Nodes are color-coded based on their significance, with red representing the most critical targets. Lines between nodes represent the predicted functional and physical associations between these proteins, which may be influenced by resveratrol treatment.

Gene mining, target prediction and network construction were performed to provide system-level understanding into the modulation of cellular metabolic activity by resveratrol in α-synuclein model of parkinsonism in human. The results depicted in Fig. 7 represent the identified genes and protein–protein interactions of the target genes related to synuclein-induced Parkinson’s disease and resveratrol activity. Exploring the relevant targets revealed 3459 alpha-synucleinopathies (AS) targets and 9957 human PD targets. Analysis revealed 3401 common targets to AS and PD representing synuclein-induced parkinsonism (SIP). Among these, 47 common targets were found to overlap with resveratrol targets (733). The protein–protein interaction (PPI) network analysis of the overlapping genes revealed 47 protein nodes connected by 108 edges, each edge representing a distinct type of interaction. With an average node degree of 4.6 and a local clustering coefficient of 0.437, the network reflects a moderately dense and interconnected system, indicative of complex functional relationships among the shared targets. The presence of diverse edge colors illustrates multiple interaction types, including direct physical binding, co-expression patterns, and shared biological pathways. Notably, several proteins emerged as central hubs within the network, suggesting their pivotal role in mediating the therapeutic impact of resveratrol on Parkinsonism and contributing significantly to network integrity and functionality. These include Cytochrome p450 1A2 (CYP1A2), Glutathione S-transferase Mu 2 (GSTM2), Glutathione S-transferase alpha 1 (GSTA1), Cytochrome p450 1A1 (CYP1A1), Cytochrome p450 1B1 (CYP1B1), Glutathione S-transferase alpha 3 (GSTA3), Glycogen synthase kinase-3 beta (GSK3B), B-cell lymphoma 2 protein (BCL2), Monoamine oxidase B (MAO-B), Monoamine oxidase A (MAO-A), Pyruvate Kinase M (PKM), Protein Kinase A catalytic subunit (PRKACA), Dopamine decarboxylase (DDC) and Insulin-like growth factor 1 receptor (IGF1R) as shown in Table 1. Computation of the topological metrics of hub genes was performed to further understand their roles in the network. Table 1 also shows the values of the topology.

**Table 1:** Topological parameters of hub targets.

Proteins	Nodes	MCC	k	CC	EPC	BottleNeck	EcCentricity	BC
Cytochrome P450 Family 1 Subfamily A Member 2	CYP1A2	132	9	19.67	22.37	2	0.207	69.56
Glutathione S-Transferase Mu 2	GSTM2	121	6	18.42	19.62	1	0.201	49.18
Glutathione S-Transferase Alpha 1	GSTA1	121	6	17.67	19.49	1	0.207	20.35
Cytochrome P450 Family 1 Subfamily A Member 1	CYP1A1	121	6	19.00	19.96	2	0.207	52.07
Cytochrome P450 Family 1 Subfamily B Member 1	CYP1B1	121	6	17.50	19.97	1		13.16
Glutathione S-Transferase Alpha 3	GSTA3	120	5		19.07	1	0.275	
Glycogen Synthase Kinase 3 Beta	GSK3B	59	14	24.67	24.48	12	0.275	248.88
B-cell Lymphoma 2	BCL2	55	15	25.50	24.43	7	0.275	323.33
Monoamine Oxidase B	MAOB	40	10	22.50	23.48	2	0.275	110.60
Monoamine Oxidase A	MAOA	40	10	22.50	23.49	7	0.275	110.60
Protein Kinase cAMP-Activated Catalytic Subunit Alpha	PRKACA	32	11	22.33	23.49	5	0.275	152.53
Pyruvate Kinase M1/M2	PKM	23	9	22.17	22.09	7		236.85
Dopa Decarboxylase	DDC	19	6	18.08	20.68	2	0.207	42.44
Insulin-Like Growth Factor 1 Receptor	IGF1R	18	5	18.5	20.64	2	0.201	
Dopamine Receptor D2	DRD2	16	6	19.42	21.72	2	0.207	35.81

NB: *MCC* maximal clique centrality, *k* degree centrality, *CC* closeness centrality, *EPC* edge percolation centrality, *BC betweenness centrality.*

The CYP1A2 has the highest Maximal Clique Centrality (MCC = 132), suggesting its involvement in multiple interconnected pathways. The GSK3B and BCL-2 also scored high in MCC, reinforcing their importance in the core network modules associated with parkinsonism. The BCL-2 exhibited the highest degree (k = 15), followed by GSK3B (14). In addition, BCL-2 (25.50) and GSK3B (24.67) exhibited the highest closeness scores, indicating their central role in quickly transmitting signals through the network. Also, GSK3B (12) and BCL-2 (7) had high bottleneck values, indicating that, they may serve as key bridges between different pathways involved in neurodegeneration and neuroprotection. The MAO-B and MAO-A also had high degrees (10), emphasising their role in dopamine metabolism, which is crucial in Parkinson’s disease. The BCL-2, GSK3B, MAO-B, and MAO-A showed relatively low eccentricity scores (0.275), suggesting they are central nodes with easy access to other nodes, vital for maintaining network stability. The BCL-2 had the highest betweenness score (323.33), followed by GSK3B (248.88). The topological metrics indicated that, most of the hub genes may act as major intermediaries, controlling significant information flow related to cell survival, apoptosis, and signaling pathways in Parkinson’s disease.

### Cellular, biochemical and molecular functions of promising target proteins

**Fig. 8:**
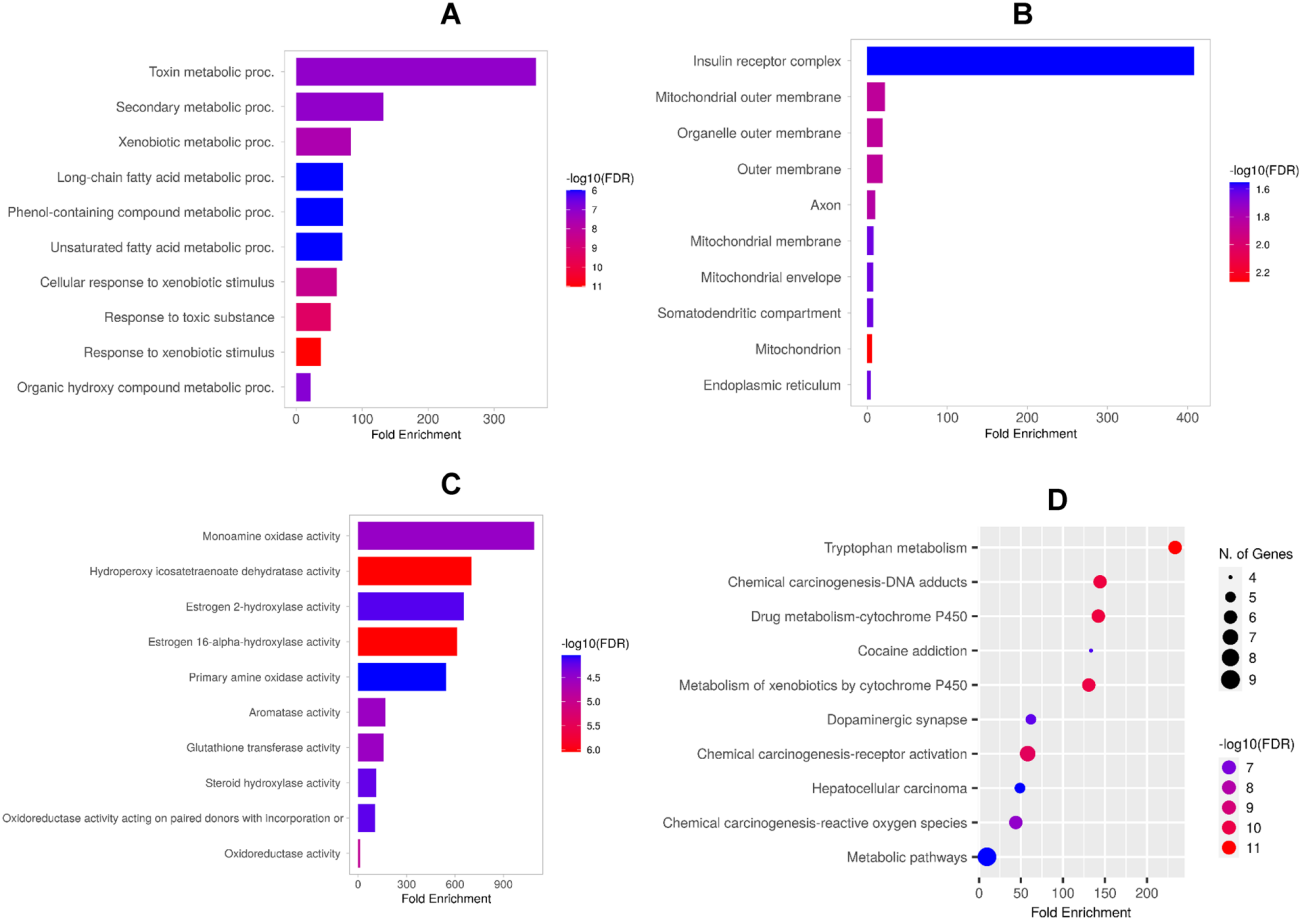
Gene Ontology (GO) and KEGG Pathway Enrichment Analysis of Resveratrol Targets in an α-Synuclein Model of Parkinson’s Disease. (**A**) Biological Process Enrichment. (**B**) Cellular Component Enrichment. (**C**) Molecular Function Enrichment (**D**) KEGG Pathway Enrichment. The dot size represents the number of genes involved in each pathway, and the color scale indicates the -log10(FDR), with darker colors representing more statistically significant pathways.

**Fig. 9:**
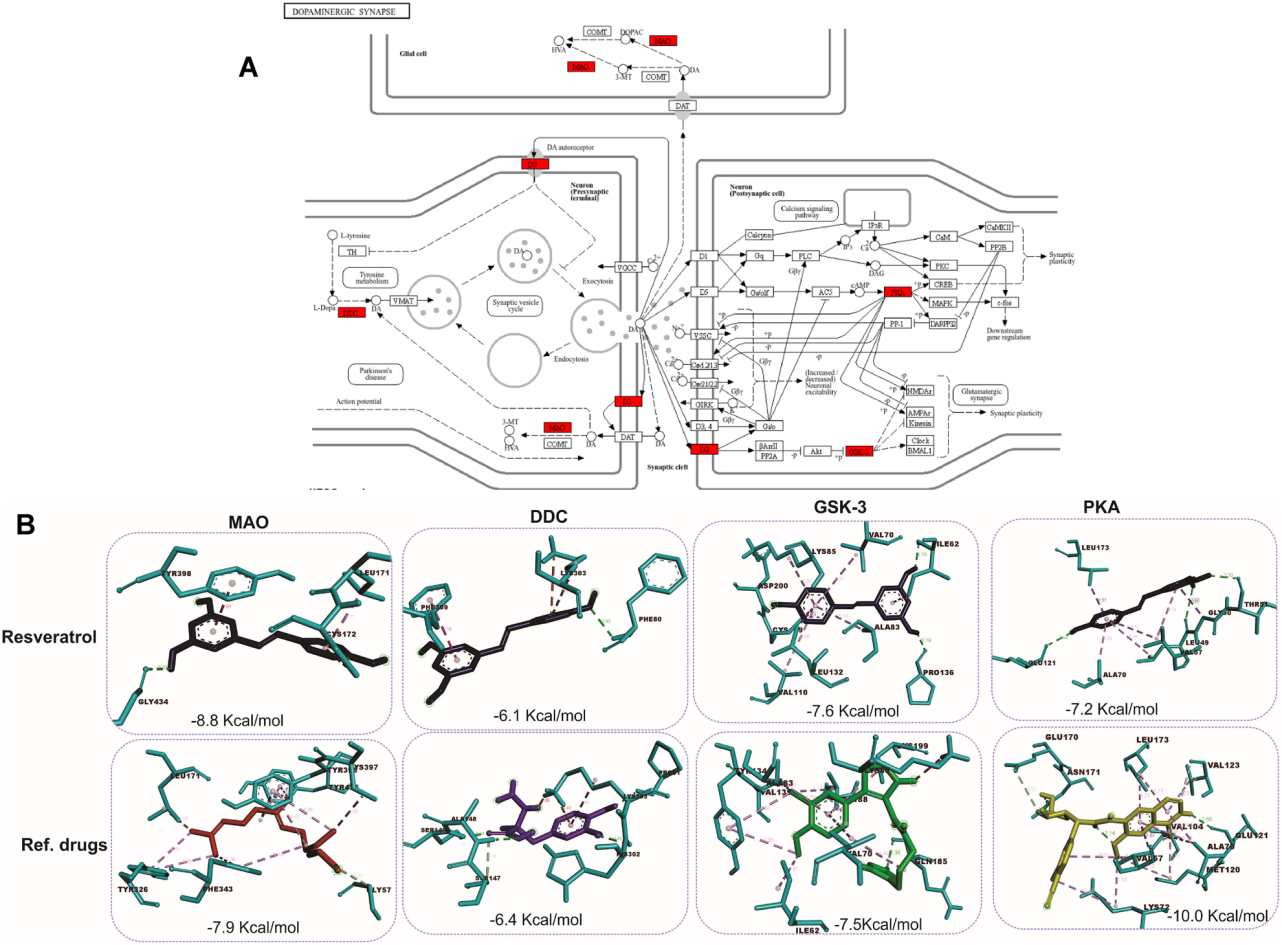
Network pharmacology of resveratrol in the dopaminergic synapse and molecular docking interactions with key enzymes. (**A**) Schematic representation of the dopaminergic synapse. The red-highlighted enzymes (MAO, DDC, GSK-3, PKA) are key targets involved in dopamine synthesis, metabolism, and signaling. These enzymes are associated with the pathology of Parkinson’s disease. (**B**) Molecular docking results of resveratrol and reference drugs with the enzymes Monoamine Oxidase (MAO), Dopa Decarboxylase (DDC), Glycogen Synthase Kinase 3 (GSK-3), and Protein Kinase A (PKA). Docking scores (kcal/mol) are shown for resveratrol (top) and reference drugs (bottom).

The Gene Ontology (GO) and pathway enrichment analysis was performed to explore the enrichment of the hub genes in various biological and biochemical processes as depicted in Fig. 8. Various biological processes including toxin metabolic process, secondary metabolic process, xenobiotic metabolic process are highly enriched (Fig. 8A). The cellular component enrichment analysis of the hub genes showing specific cellular location where these hub genes are active is represented in Fig. 8B. The various cellular structures involved in apoptosis, mitochondrial function, and detoxification are highly enriched. These include insulin receptor complex, mitochondrial outer membrane, organelle outer membrane, outer membrane, axon, somatodendritic compartment and endoplasmic reticulum. The result presented in Fig. 8C highlights key enzymatic and metabolic activities that are significantly enriched in relation to the hub genes. These biochemical functions reflect key enzymatic activities regulating neurotransmitter metabolism, lipid metabolism, estrogen metabolism, and detoxification pathways. These include the monoamine oxidase activity, hydroperoxyicosatetraenoate dehydratase activity, estrogen 2-hydroxylase activity, estrogen 16-alpha-hydroxylase activity, primary amine oxidase activity, aromatase activity, glutathione transferase activity, steroid hydroxylase activity, oxidoreductase activity acting on paired donors with incorporation of molecular oxygen and oxidoreductase activity. Furthermore, the Kyoto Encyclopedia of Genes and Genomes (KEGG) pathway enrichment analysis displayed in Fig. 8D revealed several key pathways significantly enriched based on the hub genes. These include tryptophan metabolism, chemical carcinogenesis—DNA adducts, drug metabolism—Cytochrome P450, cocaine addiction, metabolism of xenobiotics by cytochrome P450, dopaminergic synapse, chemical carcinogenesis—receptor activation, hepatocellular carcinoma, chemical carcinogenesis, reactive oxygen species and metabolic pathways (Fig. 8D). The pathway analysis of the hub genes highlights the important roles of MAO, DDC, D2, PKA, GSK-3 in the dopaminergic synapse and its dysfunction as depicted in Fig. 9A.

### Molecular docking and interactions of resveratrol with target proteins

**Fig. 10:**
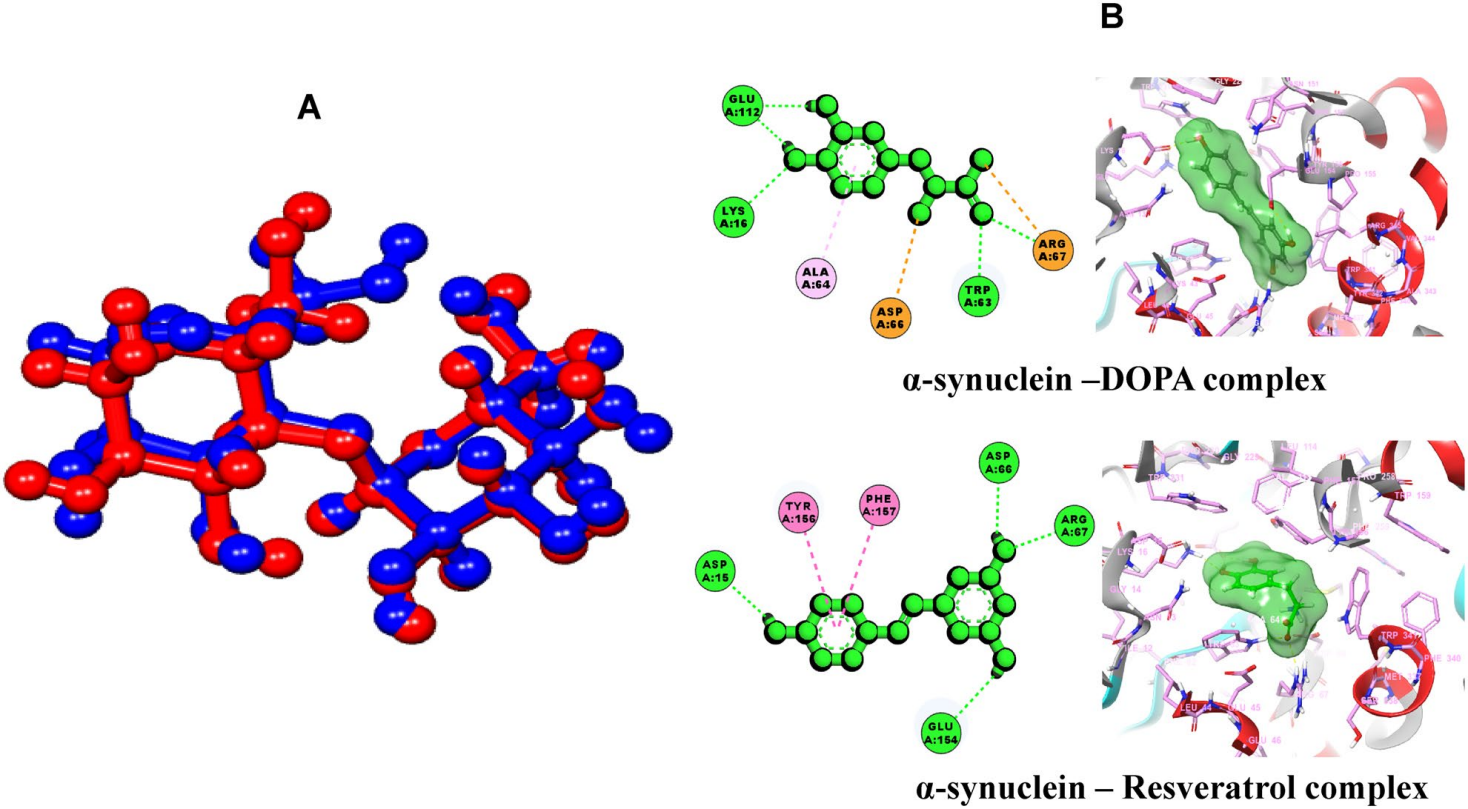
(**A**) α-synuclein co-crystalized ligand super-imposed with its docked pose (**B**) 2D and 3D interactions of lead compounds with the amino acid residues of the target binding pocket.

The docking results shown in Fig. 9B demonstrate the binding affinities of resveratrol compared to reference drugs for selected proteins involved in the dopaminergic synapse pathway, specifically MAO, DDC, GSK-3, and PKA. These proteins play significant roles in Parkinson’s disease (PD), and the docking scores (measured in kcal/mol) represent the interaction energy, where lower (more negative) values suggest stronger binding affinities. Next, we investigated the docking interactions of resveratrol with α-synuclein. The docking protocol was validated by molecular docking analysis with RMSD at 1.009 Å as shown in Fig. 10A. In docking, resveratrol and L-DOPA revealed varying degrees of binding affinities for α-synuclein with L-DOPA having the lowest docking score of − 9.826 kcal/mol and MM/GBSA of − 36.826 compared to resveratrol with a docking score of − 6.861 kcal/mol and MM/GBSA of − 36.470 (Table 2). The result revealed that the reference drug L-DOPA has a better binding affinity than the bioactive compound (resveratrol). The compounds interacted with the amino acid residues of α-synuclein binding pockets with various interactions such as hydrogen bonds, van Dal Waal interactions, pi-pi stacked etc. As shown in Fig. 10B, resveratrol showed four hydrogen bond interactions with ASP 15, GLU 154, ARG 67, and ASP 66 while DOPA showed five hydrogen bond interactions with ARG 67, TRP 62, GLU 112, and LYS 16. Figure 10B shows the 2D and 3D interaction as revealed by protein–ligand complexes.

**Table 2:** Molecular docking and MM/GBSA analysis of compounds with 3Q25.

Entry name	XP docking score (kcal/mol)	MM/GBSA ∆G bind	No of H-bond	Interacting residues
Resveratrol	− 6.861	− 36.470	4	ASP 15; GLU 154; ARG 67; ASP 66
DOPA	− 9.826	− 36.826	5	ARG 67; TRP 62; GLU 112; LYS 16

#### Results of the quantum chemical calculation

**Fig. 11:**
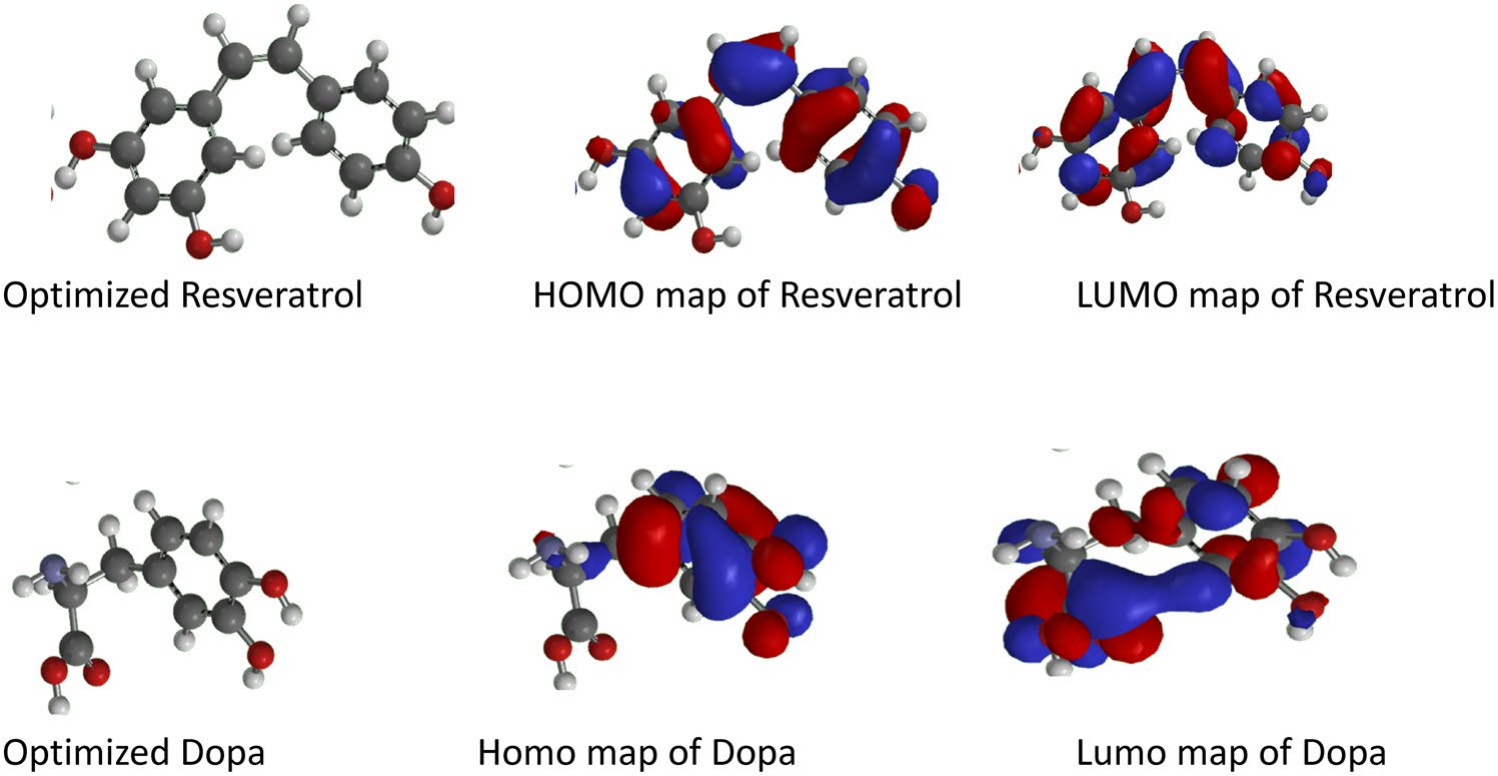
The optimized structure, HOMO and LUMO of Resveratrol and LDOPA.

As shown in Fig. 11, the high occupied molecular orbital energy (E_HOMO_) values of resveratrol and DOPA are − 5.6 and − 5.42 eV, respectively, while the low unoccupied molecular orbital energy (E_LUMO_) values are − 0.07 and − 1.02 eV, respectively. The energy band gaps (Eg) were calculated to vary from 5.53 to 4.40 eV. Table 3 shows the results of other descriptors investigating the stability and reactivity of the compounds such as chemical hardness (η), softness (δ), electronegativity (χ), and chemical potential (Cp) as studied parameters. The chemical hardness and softness values for resveratrol and DOPA range from 2.765 to 2.20 eV and 0.362 to 0.454 eV^−1^, respectively. The electronegativity values for resveratrol and DOPA were − 5.67 and − 6.44 eV, respectively.

**Table 3:** Molecular properties obtained via DFT.

Compounds	E_HOMO_ (eV)	E_LUMO_ (eV)	E_g_ (eV)	η (eV)	δ (eV^-1^)	χ (eV)	C_P_ (eV)
Resveratrol	− 5.6	− 0.07	5.53	2.765	0.362	− 5.67	5.67
DOPA	− 5.42	− 1.02	4.4	2.2	0.454	− 6.44	6.44

η = chemical hardness, δ = softness, χ = electronegativity and C_p_ = chemical potential.

## Discussion

The use of model organisms with genetic homology to humans, such as *Drosophila melanogaster,* enables the investigation of molecular mechanism of PD, offering optimal alternative and complementary platforms for the evaluation of disease etiology and pathology as well as potential therapeutic agents. *Drosophila melanogaster* serves as a valuable model for investigating both familial and sporadic variants of PD. A widely used method for gene manipulation in this organism is the yeast-derived UAS/GAL4 system (upstream activation sequence/Gal4), a highly effective bipartite technique that enables targeted gene expression^44^. Using this approach, several transgenic models of *Drosophila* are available for studying various diseases. Flies expressing wild-type or mutated forms of *SNCA* genes exhibit analogous pathologies to those inherent to PD patients such as reduced climbing ability, shortened lifespan and the presence of protein inclusions similar to Lewy bodies^45^. Higher mortality has been reported in PD, and interventions that extend lifespan can protect against this disease^46^. Previously, studies have shown that resveratrol has lifespan-extending properties in wild-type *D. melanogaster*^36^. Flies treated with resveratrol exhibited a significantly higher survival rate compared to untreated PD models, likely mediated by the activation of genes such as Sirtuin 1 and AMP-activated protein kinase (AMPK)^47^.

Resveratrol was reported to attenuate several deficits in various animal models of neurodegenerative diseases^48^. For instance, one of the hallmarks of PD is movement disorder, which we evaluated in this study using the climbing activity assay. A reduced climbing rate observed in the PD flies may suggests a reduction in dopamine content in the brain, due to SNCA aggregation-induced dopaminergic neurodegeneration^49^. This leads to the accumulation of oxidative stress markers and depletion of antioxidants markers^49^ as noted in this study. An improved climbing rate in PD flies after exposure to resveratrol-containing diet points to its efficacy in PD flies. Since L-DOPA is a dopamine replacement therapy, an improved climbing rate was also observed in PD flies treated with L-DOPA.

Acetylcholine is an essential neurotransmitter found in the brain and neuromuscular junctions^50^ and is broken down by acetylcholinesterase to acetate and choline, thus terminating the process of neurotransmission. A strong relationship exists between the cholinergic system and neurodegenerative disease. Thus, a reduction in acetylcholinesterase activity as observed in this study can have significant adverse effects on the various physiological and behavioural aspects of the flies. However, resveratrol normalizes this condition consistent with its neuroprotective properties.

Oxidative stress, a result of an imbalance between mitochondria-generated Reactive Oxygen Species (ROS) such as hydrogen peroxide and superoxide anion (O_2_^−^), and antioxidant molecules, has been implicated in various neurodegenerative diseases^51,52^. The brain is inquisitively susceptible to ROS as it consumes the largest portion of oxygen and it contains high amounts of lipids and transition metals involved in the generation of hydroxyl radicals and other ROS^53^. There is indirect evidence providing a free radical hypothesis for neurodegeneration in PD^54^. Dopamine oxidation and overstimulation of excitatory amino acid receptors such as glutamate receptors cause overproduction of reactive oxygen species like hydrogen peroxide, dopamine quinones, and nitric oxide^54^.

The hallmarks of PD are characterized by Lewy neurites and Lewy bodies. Nitric oxide is a short-lived, important signalling molecule in the central nervous system^55^. In physiological conditions, it regulates several biological processes including neuronal cell development^55^. It is also involved in different brain-related diseases^56^. Elevated levels of NO can lead to the generation of peroxynitrite in the presence of hydrogen peroxide and superoxide radicals^57^. Nitration of the SNCA protein through tyrosine oxidation induces its aggregation to form Lewy bodies^58^. The pathophysiological consequence of oxidation of the lipids by ROS is lipid peroxidation^59^. The observed elevated levels of nitric oxide and MDA (a marker of lipid peroxidation) in the *Drosophila* SNCA model of PD, were mitigated by resveratrol.

Glutathione is a component of the non-protein thiols. It is an antioxidant molecule that responds to elevated free radicals and ROS production^60^. Paik et al.^61^ observed decreased levels of glutathione (GSH) in PD. They also observed a direct molecular interaction between α-synuclein aggregation and GSSG as a possible consequence of reduced GSH level and the increased hydrogen peroxide (H_2_O_2_) generation in PD. The observed decrease in non-protein thiols and the increase in total hydroperoxide levels in the PD flies corroborate the findings of Paik^61^. Here, we found that resveratrol ameliorated PD-induced accumulation of total hydroperoxide and reduction in non-protein thiols. Catalase is one of the most active enzymes converting hydrogen peroxide to water and molecular oxygen^62^. Catalase activity decreased in the brains and plasma of Alzheimer’s disease (AD) patients^63^, and the basal nuclei of PD patients^64^. The decrease in the activity of catalase in PD flies possibly triggered the accumulation of total hydroperoxide. Resveratrol, however, attenuated the PD-induced decrease in catalase activity in PD flies. The cellular thiol redox state is an important factor for different metabolic processes, thus a shift in the redox state causes oxidative stress^65^. Total thiol constitutes all sulfhydryl-containing molecules, and they are important in maintaining the cellular redox state. The reduction in the levels of total thiol and non-protein thiols in PD flies was mitigated following treatment with resveratrol.

Superoxide dismutase 1 (SOD) is encoded by the *Sod 1* gene in *D. melanogaster.* It is an innate antioxidant defence system that protects the living system against ROS by degrading superoxide radicals (O_2_^−^) to molecular oxygen (O_2_) and hydrogen peroxide (H_2_O_2_), thereby providing peroxide for further breakdown by catalase^37^. Thus, in this study, gene expression analysis was used to determine the expression of the *Sod 1* gene. Here, we observed downregulation in mRNA expression of *sod 1* in PD flies, while treatment with L-DOPA and resveratrol (15, 30, and 60 mg/kg diet) significantly upregulated the gene when compared with PD flies, thus corroborating the adverse effect of oxidative stress in PD flies^66^. The enzymatic and non-enzymatic antioxidant molecules are crucial defense systems against ROS and RNS, and they basically catalyze the elimination of reactive species from biological system. Antioxidants such as SOD and catalase are responsible for the metabolism of ROS and RNS generated during mitochondria respiration^67^. However, a defect in the mitochondria respiration pathway may enhance the level of ROS and RNS, which may overpower the capacity of the antioxidant defense system. Studies showed that in PD, the vast majority of the O_2_^-^ is produced by the mitochondria complex I and III, which is reduced to H_2_O_2_ by SOD^68^. Therefore, the observed decrease in *sod1* mRNA level may reduce its defense system against detrimental effects of O_2_^−^.

Since the pathophysiology of PD has been linked to mitochondrial dysfunction^69^, we carried out cellular metabolic activity assay using 3-(4,5-dimethylthiazol-2-yl)-2,5-diphenyl tetrazolium bromide (MTT). Indeed, accumulation of α‐synuclein, mitochondrial dysfunction, and mis-regulation of gene expression reduce cell viability and ultimately cell death in PD^70^. Understanding the mechanisms of mitochondrial function in PD could aid the development of drugs targeted to preserve cell viability and monitor PD progression in patients^71^. For instance, the protective effects of various compounds, such as erythropoietin and arachidonic acid, prevented cell death in different PD models^72^. Here, we found that resveratrol (15, 30 and 60 mg/kg diets) ameliorated the reduction in cellular metabolic rate in *Drosophila melanogaster* after exposure to PD flies*.*

Network pharmacology was applied to provide insight into the therapeutic potential of resveratrol in mitigating parkinsonism induced by synucleinopathies as well as its underlying mechanism in human. The identified common targets (47) revealed a set of shared targets/pathways that are crucial for both the pathogenesis of SNCA-induced PD and the therapeutic potential of resveratrol. This supports the notion that resveratrol can as well provide beneficial effects in humans as reported in the *Drosophila* α-synuclein model of Parkinson’s Disease. The diversity of interactions (physical, co-expression, genetic interactions, etc.) among the selected protein targets in the PPI network suggests a multi-faceted approach to regulation and impact of resveratrol. It revealed the interconnectedness of the Cytochrome P450 enzymes (CYP1A2, CYP1B1, CYP1A1), Monoamine Oxidases (MAO-A and MAO-B), GSK3B, BCL-2, Pyruvate Kinase M (PKM) and Protein Kinase A catalytic subunit (PRKACA). The topology metrics of the hub genes in the PPI network provided valuable insights into the importance of these genes within the network. These hub genes, particularly BCL-2, GSK3B, MAO-B, and MAO-A are critical players in the network as indicated by their topological metrics highlighting their central roles in apoptosis regulation, dopamine metabolism, and neuroprotection, underscoring the molecular mechanisms through which resveratrol might exert its beneficial effects in Parkinson’s disease. The high degree, closeness, and betweenness of BCL-2, a well-known protein that regulates apoptosis, suggest its pivotal role in preventing apoptosis, making it a potential target for resveratrol’s neuroprotective actions. The value of degree is the number of direct connections a node has with other nodes where a higher degree indicates more interactions, signifying that the gene is a central hub in the network. The closeness shows the reciprocal of the sum of the shortest path distances from the node to all other nodes, indicating that the gene can quickly influence other nodes, making it efficient in signal transmission. High betweenness value, which indicates the number of times a node acts as a bridge along the shortest path between two other nodes suggest the gene’s role in controlling the flow of information through the network. The GSK3B, is another important hub gene in the network as it shows high values for most topological metrics indicating a critical hub, especially in regulating pathways related to neurodegeneration and cell survival. The protein is extensively reported for its role as a critical kinase involved in various signaling pathways, including those regulating neurogenesis, inflammation, and protein phosphorylation^73–75^. The MAO-B and MAO-A with moderate degree and high closeness emphasize their importance in dopamine metabolism in human, an essential pathway that may be affected by resveratrol in Parkinson’s disease. *Drosophila* only displays MAO-like activity, as this enzyme is not present in the flies. The critical roles of these enzymes in parkinsonism are widely reported as they are mainly involved in the degradation of neurotransmitters such as dopamine^76–78^. The inhibition or modulation of these enzymes can protect against neurotoxicity and reduce oxidative stress.

The biological and cellular functions of the hub genes were explored in this study to further understand the role of the identified target proteins in various cellular metabolic activity and mechanism that may underpin the pathogenesis of synuclein-induced Parkinson’s disease and the therapeutic potential of resveratrol as indicated by the study. Enrichment of the hub genes in various biological processes as reported in this study (Fig. 8) suggests that the hub genes are central to detoxification, oxidative stress reduction, fatty acid metabolism, and apoptosis prevention. Toxin metabolic process, the top biological process enriched involves the metabolism of harmful substances, which is crucial in neurodegenerative diseases like Parkinson’s. The GSK3B and MAO-B may be involved in such process related to oxidative stress, which is often triggered by toxins. Resveratrol, by influencing these genes, may help reduce oxidative damage in neurons. Secondary metabolism also enriched often involves the synthesis and degradation of compounds like neurotransmitters. In Parkinson’s disease, the metabolism of dopamine and other neurotransmitters is dysregulated. Xenobiotic metabolic process is also highly enriched as shown in the results. Xenobiotics are foreign compounds, and their metabolism is crucial in protecting cells from external toxins. The cytochrome P450 system including the CYP1A2, CYP1B1, CYP1A1 is key in detoxifying harmful compounds, especially those that contribute to oxidative stress in neurons^79^. Furthermore, the hub genes were suggested in this study to be enriched and located in key cellular structures involved in apoptosis, mitochondrial function, and detoxification. These components are vital in neurodegeneration, and the beneficial action of resveratrol may help to enhance mitochondrial health, prevent apoptosis, and reduce oxidative stress. In synucleinopathies such as Parkinson’s disease, mitochondrial dysfunction plays a critical role in disease progression as the mitochondrial outer membrane is critical in apoptosis regulation and mitochondrial function^80,81^. The BCL-2 identified as a hub gene in this study is an anti-apoptotic protein that helps maintain mitochondrial integrity by preventing the release of pro-apoptotic factors^82,83^. Resveratrol may have an impact on BCL-2 activity towards enhancing mitochondrial health, which is crucial for protecting neurons in Parkinson’s disease. Enrichment of the axon as suggested may involve GSK3B which plays a critical role in axonal maintenance and repair as BCL-2 prevents axonal degeneration by inhibiting apoptosis^84^. Possible regulation of these genes by resveratrol may enhance axonal health and function, which is crucial for maintaining neural networks affected by Parkinson’s disease. In addition, the enriched somatodendritic compartment including the cell body (soma) and dendrites of neurons, where signals are received and processed may also involve GSK3B’s role in synaptic plasticity and dendritic maintenance^85^.

The biochemical and molecular enrichment of the hub genes indicate significant enzymatic and metabolic activities which center around key enzymatic activities that regulate neurotransmitter levels (monoamine oxidase and primary amine oxidase activities), protecting against oxidative stress (glutathione transferase and oxidoreductase activities), modulating estrogen metabolism, which may have neuroprotective effects and reducing inflammation and lipid peroxidation through enzymes involved in fatty acid metabolism. By targeting these molecular pathways, resveratrol may help preserve neuronal function and slow down the progression of neurodegeneration in Parkinson’s disease. The enriched KEGG pathways highlight critical biological processes related to metabolism, detoxification, dopamine signaling, and oxidative stress, all of which are relevant to Parkinson’s disease. Resveratrol’s potential benefits in this model may stem from its ability to modulate neurotransmitter pathways (especially dopamine), enhance detoxification processes (via cytochrome P450), and reduce oxidative stress. Enrichment of the dopaminergic pathway is particularly relevant to Parkinson’s disease, as it involves dopamine signaling, a key neurotransmitter that is severely depleted in the disease. Resveratrol’s modulation of dopaminergic synapses could contribute to restoring dopamine levels and improving neural communication. Further KEGG pathway analysis shows that the hub genes MAO, DDC, D2, PKA, GSK-3 play key roles in dopamine metabolism, signaling, and neuroprotection in the dopaminergic synapse. The beneficial effects of resveratrol in the model of Parkinson’s disease are likely mediated through its interaction with these pathways. The DDC is responsible for converting L-DOPA into dopamine in the presynaptic neuron^86,87^. In PD, the loss of dopaminergic neurons reduces the efficiency of dopamine production. L-DOPA, the precursor to dopamine, is often administered as a treatment to enhance dopamine levels. The D2 Receptor are postsynaptic receptors involved in modulating the release and synthesis of dopamine. They are critical in both motor and cognitive functions. Dysregulation of D2 receptor activity is a hallmark of PD, leading to impaired dopaminergic transmission and motor control issues^88^. The PKA is an enzyme involved in the phosphorylation of various proteins, which is crucial for intracellular signaling, including dopamine-related pathways. Altered PKA signaling can disrupt cellular processes associated with dopamine release and neuroplasticity, leading to PD progression. Resveratrol may modulate PKA activity, potentially restoring proper signaling within dopaminergic neurons and aiding in synaptic plasticity, which could help mitigate PD symptoms. Overactivation of GSK-3 has been linked to neurodegeneration in PD, contributing to neuronal death and the misfolding of α-synuclein^89^. Resveratrol is known to inhibit GSK-3 in colon cancer^90^, and may also protect neurons from apoptosis and reduce α-synuclein aggregation, thus providing a protective effect in PD models. Taken together, inhibiting MAO and GSK-3, enhancing dopamine receptor activity, and possibly influencing PKA signaling, resveratrol may reduce oxidative stress, prevent neurodegeneration, and improve dopaminergic function in the context of PD.

Based on the prominent roles of MAO, DDC, GSK-3 and PKA in the dopaminergic signaling involved in the beneficial role of resveratrol in parkinsonism as suggested in this study, their interactions with resveratrol were clarified using molecular docking. In addition, interaction of resveratrol with α-synuclein was explored. The molecular docking analysis of protein targets and small molecules predicts the binding interaction and conformation for a defined binding site of the target. It is a vital tool in drug design and development^91^. The primary data from molecular docking is the binding energy, which expresses the strength and affinity of the target-ligand interaction. Therefore, the more negative the binding energy value, the stronger the receptor-ligand interaction^92^. The results showed that, resveratrol exhibited strong inhibitory potential against GSK-3; and may be a stronger inhibitor than the reference drug. It showed strong binding affinity to GSK-3, making important molecular contacts LYS85, ASP200, and CYS199. The reference drug binds similarly to GSK-3 with a comparable affinity interacting with residues like ILE62 and VAL70, suggesting that both Resveratrol and the reference drug are effective GSK-3 inhibitors. The catalytic triad of GSK-3β typically consists of three key amino acids involved in catalysis: Lys85, Glu97, and Asp200^93^. These residues are crucial for the enzyme’s function, facilitating the transfer of a phosphate group to target substrates during phosphorylation. The Lys85 plays a crucial role in positioning ATP and stabilizing the phosphate group for transfer to the substrate while Glu97 functions as a proton acceptor, helping in the activation of the substrate for phosphorylation by facilitating the deprotonation of the substrate’s hydroxyl group^94^. The Asp200 participates in the coordination of the metal ion (often Mg^2^⁺) that is required for the phosphorylation reaction, ensuring proper alignment of ATP and stabilizing the reaction intermediates. The catalytic triad is essential for the kinase activity of GSK-3β, enabling it to regulate a wide range of signaling pathways, including those involved in cell survival, metabolism, and neurodegenerative diseases like Parkinson’s. Strong binding of resveratrol with MAO was also reported in this study. Resveratrol has a strong inhibitory potential against MAO, comparable to the reference drug, making it a potential agent for reducing dopamine breakdown in PD. It shows a strong binding affinity to MAO, with several key interactions, notably with TYR398, GLY434, and LEU171. This indicates that resveratrol could effectively inhibit MAO, reducing dopamine degradation and potentially alleviating PD symptoms by increasing dopamine availability. The reference drug binds similarly to resveratrol, interacting with TYR326 and PHE343, but with a slightly lower binding affinity. This suggests resveratrol may be comparable or slightly superior to the standard drug in inhibiting MAO. Resveratrol exhibits moderate binding to DDC, a key enzyme in converting L-DOPA to dopamine. Interactions occur with residues like PHE80 and LEU503, but the binding is not as strong as other proteins, indicating that the inhibitory potential of resveratrol against DDC might be limited as the reference drug shows a slightly stronger affinity than resveratrol, forming more interactions with ALA148 and ALA147, which could imply it is more efficient at modulating DDC activity. Resveratrol also shows lower affinity binding for PKA than the reference drug. Taken together, the docking results suggest that resveratrol could be particularly beneficial for its inhibitory action on MAO and GSK-3, providing neuroprotection by enhancing dopamine availability and preventing neuronal apoptosis. However, it appears less effective in interacting with DDC and PKA compared to the reference drugs.

To investigate the molecular interactions of resveratrol and L-DOPA within the binding site of α-synuclein, extra precision (XP) Glide docking and molecular mechanics/generalized Born surface area (MM/GBSA) calculations were carried out. Prior to docking, the reliability of the docking protocol was validated by removing the co-crystallized ligand from the target protein structure, preparing it, and re-docking it into the same active site. The resulting root mean square deviation (RMSD) between the original and re-docked conformations was 1.009 Å, which falls below the acceptable threshold of 2 Å, confirming the accuracy and reproducibility of the docking method^95^. The co-crystalized ligand is blue in colour and the re-docked ligand is red. The binding energy of LDOPA (− 9.826 kcal/mol) was observed to be lower compared with resveratrol (− 6.861 kcal/mol). This suggests that the reference drug (LDOPA) exhibited higher binding affinity with the α-synuclein active site compared with the active compound (resveratrol). The result of the analysis showed that the compounds made molecular contact with the amino acid residues of the binding site. Resveratrol was observed to interact with ASP 15, GLU 154, ARG 67, and ASP 66 using five (5) hydrogen bonds while DOPA interacted with ARG 67, TRP 63, GLU 112, and LYS 16 using four (4) hydrogen bonds. The hydrogen bond interaction of small molecules with the amino acid residues at the catalytic site of protein is vital for their modulatory efficacy^91^. The formation of H-bonds between the ligands and active site residues of α-synuclein might be associated with the presence of functional groups on the structures of the ligands. DOPA formed five (5) hydrogen bonds with the hydroxyl group while resveratrol formed four (4) hydrogen bonds with the hydroxyl group, three (3) hydrogen bonds with the carbonyl group, and one with the amino group of the compound.

The density function theory (DFT) describes the interaction and overlapping of molecules to show the ability of small molecules to transfer electrons. Therefore, the higher occupied molecular orbital (HOMO) and low unoccupied molecular orbital (LUMO) energies are used to predict the reactivity of small molecules^96^. The E_HOMO_ value of resveratrol (− 5.60 eV) and E_LUMO_ value of − 0.07 eV indicate that resveratrol might readily donate electrons and possess high reactivity compared with L-DOPA with E_HOMO_ and E_LUMO_ values of − 5.42 eV and − 1.02 eV respectively. Since higher values of E_HOMO_ and lower values of E_LUMO_ are responsible for the increased reactivity of molecules. Other reactivity descriptors (E_g_, η, δ, χ, and C_P_) showed that resveratrol is more stable than L-DOPA. These parameters have been reported as indicators of chemical stability and reactivity of molecules^97^.

**Scheme 1:**
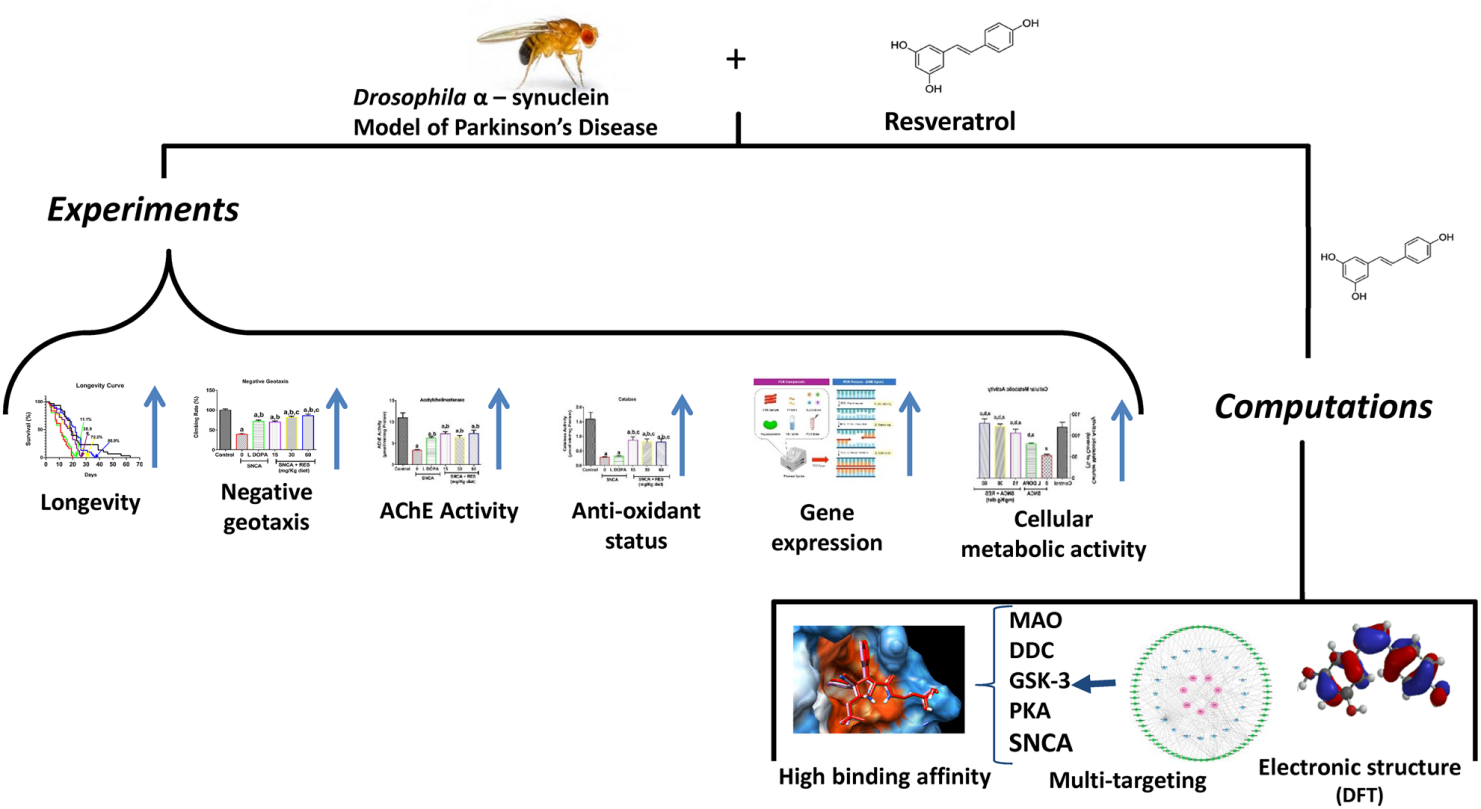
Beneficial mechanisms of action of resveratrol in *Drosophila melanogaster* model of Parkinson’s disease.

Overall, this study examined the efficacy of resveratrol at different concentrations in transgenic α-synuclein model of PD in *Drosophila melanogaster*. Resveratrol improved behavioural function, antioxidant biomarkers, and reduced markers of oxidative stress in PD flies. Resveratrol also increased the cellular metabolic activity and mRNA level of the *sod1* gene (Scheme 1). The hub genes identified, which include GSK3B**,** BCL2**,** MAO-B**,** CYP1A2**,** and GSTM2 represent critical nodes within the protein network involved in the therapeutic potential of resveratrol in PD. The study suggests that the hub genes are involved in essential processes such as dopamine metabolism, apoptosis, oxidative stress, and cell survival. Resveratrol could be particularly beneficial for its inhibitory action on MAO and GSK-3 as indicated by the prominence and connectivity of these target proteins in the protein–protein interaction network and their strong binding interactions with resveratrol. Furthermore, resveratrol bound and interacted with specific amino acids at the binding pocket of human alpha-synuclein possibly inhibiting its aggregation while the density functional theory revealed that resveratrol is a good donor and acceptor of protons. Therefore, resveratrol may be useful in the management of PD. Future perspectives in the field would be to understand if resveratrol could be used in clinical trials in conjunction with the available drugs to understand if it could alleviate the oxidative stress associated with PD in patients. It would also be interesting to study the possible beneficial role of resveratrol in other fly models of PD such as *pink-1* loss of function.

## Materials and methods

### Chemicals

Reduced glutathione (GSH), 1-chloro- 2,4-dinitrobenzene (CDNB), acetylthiocholine iodide and 5′,5′-dithiobis(2- nitrobenzoic acid) (DTNB) were purchased from Sigma Aldrich (St. Louis, MO, USA).

### *Drosophila melanogaster* culture

*Drosophila melanogaster* stocks and crosses were maintained and reared on cornmeal medium mixed with brewer’s yeast (1% w/v), agar–agar (1% w/v), and nipagin (preservative, 0.08% v/w) at temperature (23 ± 2 °C) under 12 h dark/light cycle in the *Drosophila* Laboratory, Department of Biochemistry, University of Ibadan, Oyo-State, Nigeria.

### Crossing of flies

Flies harbouring the human α-synuclein transgene (UAS-SNCA/CyO) and the pan-neuronal driver neuronal-synaptobrevin (nSyb)-Gal4 were used in this study, with *w*^*1118*^ strain used as background control. Virgin females of nSyb-Gal4 flies were crossed with males of the UAS-SNCA flies for 5 days. The parent flies were removed, and the non-balancer offspring (F1) were used for the experiments. Both male and female flies (1–3 days post-eclosion) in which wild-type human α-synuclein was overexpressed in neurons were used in all experiments.

### Treatment of flies and preparation of samples

Newly eclosed flies from the control and PD groups were placed in culture vials (30 flies per vial) containing the different concentrations of resveratrol. The longevity assay was carried out with both genders of the flies (1- to 3 days old) as previously described^36^*.* The flies were divided into different groups each containing five replicates/group (30 flies/vial). They were fed with diet supplemented with resveratrol (15 mg/kg diet (approximately 6.57 mM); 30 mg/kg diet (approximately, 13.14 mM) and 60 mg/kg diet (approximately, 26.28 mM) for a lifespan. Resveratrol was first dissolved in the vehicle (absolute ethanol, final concentration of 2% in the diet). The PD flies were also exposed to L-DOPA (200 µL of ﻿1.0 mg/mL mixed in 10 g diet (0.1 mM)). Daily mortality was recorded and used to plot the survival curve. Similar concentrations of resveratrol were administered via the diet to PD flies for 21 days to evaluate its possible beneficial action. Afterwards, flies were anesthetized under CO_2_, weighed, homogenised in 0.1 M phosphate buffer (pH 7.4, ratio of 1 mg:10 µL), and centrifuged at 4000*g* for 10 min at 4 °C in a refrigerated centrifuge (Thermo Fisher Sorvall Legend Micro 17R (Fresco). Thereafter, supernatants were transferred into newly labelled microfuge tubes and used for the determination of total hydroperoxides, which included hydrogen peroxide (H_2_O_2_), total thiol (T-SH), non-protein thiols, nitric oxide (NO, nitrate/nitrate) levels, as well as catalase, glutathione-S-transferase (GST), and acetylcholinesterase (AChE) activities.

### Evaluation of negative geotaxis assay

The locomotor performance (negative geotaxis) of PD flies was evaluated as previously described^36^*.*

### Determination of biochemical parameters

#### Determination of total protein level

Protein concentration was determined based on the method described by Lowry et al.^98^. Sample (25 μL, 1:10 dilution) was added to 135 μL of distilled water and mixed with 400 μL of alkaline copper sulphate reagent (Lowry reagent) followed by an incubation time of 15 min. Thereafter, Folin-Ciocalteu solution (1:5 dilution) was added. After 20 min of incubation at room temperature, the absorbance was measured at a wavelength of 660 nm against a blank in a spectrophotometer. Protein values were extrapolated from the BSA standard curve.

#### Determination of acetylcholinesterase activity

Acetylcholinesterase activity was evaluated using the method of Ellman^99^. The reaction mixture contained 135 μL of distilled water, 20 μL of 100 mM potassium phosphate buffer (pH 7.4), 20 μL of 10 mM DTNB, 5 μL of the sample, and 20 μL of acetylthiocholine (8 mM). Then, the reaction was monitored at a wavelength of 412 nm (for 5 min, 15 s intervals) in a SpectraMax microplate reader (Molecular Devices, USA). The data were expressed thereafter in μmol/min/mg protein.

#### Determination of catalase activity

The activity of catalase was determined with the method of Aebi^62^. The reaction medium contained 50 mM phosphate buffer (pH 7.4), 19 mM hydrogen peroxide, and 10 μL of sample (1:50 dilution). Subsequently, the decrease in absorbance of H_2_O_2_ (ε = 39.4 mM-1 cm-1) at a wavelength of 240 nm was recorded for 2 min (10 s interval) using a SpectraMax microplate reader (Molecular Devices). Thereafter, the activity of catalase was calculated and expressed in μmol of H_2_O_2_ consumed/min/mg of protein.

#### Determination of total hydroperoxide level

Total hydroperoxides, which included Hydrogen peroxide was carried by the use of the method of Wolf^100^. Briefly, 10 μL of the sample was added to a 590 μL of FOX containing a mixture of 100 mM of xylenol orange, 250 mM ammonium ferrous sulphate, sorbitol (100 mM), and 25 mM H_2_SO_4_. Then, the reaction mixture was incubated at room temperature for 30 min, and the absorbance was measured in a spectrophotometer at a wavelength of 560 nm. Thereafter, total hydroperoxide level (including H_2_O_2_) level was calculated and expressed in μmol/L.

#### Determination of total thiols content

The total thiol level was determined using the method of Ellman^101^. Briefly, the reaction mixture contained 270 μL of 0.1 M of potassium phosphate buffer (pH 7.4), 20 μL of sample, and 10 μL of DTNB (10 mM). This was followed by incubation at room temperature for 30 min. The absorbance was then measured at a wavelength of 412 nm using a SpectraMax microplate reader (Molecular Devices). The total thiols values were calculated using GSH standard curve and expressed in μmol/mg protein.

#### Measurement of nitrite and nitrate as indices of nitric oxide (NO, nitrite/nitrate) production

The level of nitric oxide (NO) was quantified using Griess reagent (1.5% sulphanilamide and 0.15% N-1-naphthyl-ethylene diamine in 1 N HCl), based on the enzymatic conversion of nitrate to nitrite. Samples were mixed with Griess reagent in a ratio of 1:1, and nitric oxide level (nitrite and nitrate) was quantified from sodium nitrate standard curve measured at 550 nm^102^.

#### Determination of non-protein thiols level

The non-protein thiols content was estimated colorimetrically using Ellman’s reagent (DTNB) according to the procedure described by Jollow et al.^103^. The supernatant was precipitated with 4% sulphosalicyclic acid (4%) in a ratio of 1:1. The samples were kept at 4 °C for 1 h and then subjected to centrifugation at 5000 rpm for 10 min at 4 °C. The assay mixture consisted of 200 µl of 0.1 M phosphate buffer, 50 µl of supernatant, and 50 µl of DTNB. The OD was read at 412 nm and the results were expressed as μmol of GSH/mg protein.

#### Lipid peroxidation assay (Malondialdehyde level)

The assay was performed as described by Okawa et al.^104^, The reaction mixture was made by adding 5 µL of 10 mM butylhydroxytoluene, 200 µL of 0.6.7% thiobarbituric acid, 600 µL of 1% *O*-phosphoric acid, 105 µL distilled water, and 90 µL brain homogenate. The resultant mixture was incubated at 90 °C for 45 min, and the OD was measured at 535 nm. The results were expressed as μmol/L.

#### Determination of cellular metabolic rate (cell viability) of PD *D. melanogaster* treated with resveratrol

We evaluated cellular metabolic rate (cell viability) based on enzymatic reduction of MTT (3-(4,5-dimethylthiazol-2-yl)-2,5-diphenyltetrazolium bromide) to MTT-formazan in control and PD flies at a final concentration of 5 mg/mL using the method of Abe and Matsuki^105^. The data were expressed as a percentage of the control.

### Isolation of RNA and quantitative real-time RT-PCR

The total RNA molecule was extracted from 25 mg of whole flies using Trizol reagent (TRI Reagent®, Zymol Research), adhering to the manufacturer’s protocol as previously reported^106^. Following electrophoresis, the extracted RNA was resuspended in 50 µL molecular-graded water, quantified spectrophotometrically with MaestroNanoDrop Pro (Maestro Gen), and imaged in a 2% agarose gel. Total RNA (0.3 µg) was used for cDNA synthesis in a BioRad T100 Thermal Cycler, using ProtoScript II (New Egland BioLabs) kit. The primer sequence used in this study (*Sod 1*) was obtained from GeneBank Overview (GenBank Overview (nih.gov) as previously reported (Table 4)^37^. The primers were custom synthesised by Invitrogen and were designed using the primer BLAST tool (http://www.ncbi.nlm.nih.gov/tools/primer-blast). The expression of the mRNA levels was then assessed by quantitative RT-PCR, with Alpha-Tubulin as reference gene. The primer sequences are listed below:

**Table 4:** Primer sequences.

	Forward sequence	Reverse sequence
*Sod1*	GGAGTCGGTGATGTTGACCT	GTTCGGTGACAACACCAATG
Alpha-Tubulin	TGGGCCCGTCTGGACCACAA	TCGCCGTCACCGGAGTCCAT

For the qPCR, the Luna Universal qPCR Master mix kit was utilised. The qPCR cycling conditions were initial denaturation at 95 °C for 60 s, followed by 40 cycles for 15 s at 95 °C, 60 °C for 60 s followed by dissociation curve analysis. The SYBR fluorescence was analysed using the ABI Prisms 7900HT from Applied Biosystems using the SDS 2.1. There were five replicates of every reaction, conducted independently of the groups. The dissociation curves were run between 60 and 95 °C to guarantee that only one product was amplified per reaction. Using the 2^−ΔΔΔCT^ method, the expression value for the Sod1 gene was determined.

### Network pharmacology

#### Gene mining, target prediction and network construction

Resveratrol-associated target genes were identified using the SwissTargetPrediction database (http://www.swisstargetprediction.ch, accessed on 16 September 2024) and PharmMapper (http://www.lilab-ecust.cn/pharmmapper/, accessed on 16 September 2024), based on its SMILES representation and 2D structural formula. The resulting gene targets were subsequently matched and standardized through the UniProt database (https://www.uniprot.org/, accessed on 16 September 2024), where redundant entries were also removed. Genes implicated in synucleinopathies and Parkinsonism were separately extracted from both the GeneCards database (https://www.genecards.org/, accessed on 16 September 2024) and the Online Mendelian Inheritance in Man (OMIM, http://omim.org/, accessed on 16 September 2024). For synucleinopathy-related genes, keyword searches included “synucleinopathies” and “alpha-synuclein,” limited to Homo sapiens, while for Parkinsonism, the search terms “Parkinson’s disease” and “Parkinsonism” were used. Gene identifiers from these searches were harmonized and deduplicated using the UniProt database.

To determine common targets between alpha-synuclein (AS) and Parkinson’s disease (PD), a Venn diagram was generated using the online Venn diagram tool (https://bioinformatics.psb.ugent.be/webtools/Venn/, accessed on 16 September 2024). Shared genes between resveratrol and synucleinopathy-related Parkinsonism (SIP) targets were also identified through intersection analysis. Subsequently, protein–protein interaction (PPI) networks were assembled through the STRING database (https://cn.string-db.org/cgi/input.pl)^107^. These interaction networks were visualized and analyzed using Cytoscape software (version 3.10.2)^108^ to obtain the hub genes. The cytoHubba plugin^109^ was applied to identify the top 10 hub genes, based on the Maximal Clique Centrality (MCC) algorithm. Further topological metrics—including degree (k), betweenness centrality (BC), clustering coefficient, and closeness centrality (CC)—were computed using the Analyze Network tool.

#### Gene ontology and pathway enrichment

The intersecting genes were subjected to functional annotation through Gene Ontology (GO) and Kyoto Encyclopedia of Genes and Genomes (KEGG) pathway enrichment analyses using the ShinyGO 0.77 platform (http://bioinformatics.sdstate.edu/go/, accessed on 16 September 2024)^110^, with an False Discovery Rate of < 0.05 and *p* of < 0.05 as cut-off values, displaying the top ten results.

### Molecular Modeling 

#### Binding assessment of resveratrol with selected hub targets

The key target proteins identified were subjected to molecular docking with resveratrol. The chemical structures of resveratrol and comparator compounds were obtained from the PubChem database (www.pubchem.ncbi.nlm.nih.gov) in Structure Data Format (SDF). These SDF files were converted into the mol2 format using Open Babel software^111^. Polar hydrogen atoms were assigned Gasteiger charges, while non-polar hydrogens were merged with their respective carbon atoms. As previously described^112^, the torsional flexibility and internal rotational bonds were fixed at zero. The prepared ligand files were then converted into PDBQT format, compatible with AutoDock Tools. The structures of the hub targets were retrieved from the Protein Data Bank (https://www.rcsb.org). From the downloaded crystal structures, the native inhibitors were extracted, and water molecules removed as demonstrated earlier^27^. Hydrogen atoms were subsequently added using AutoDock version 4.2. Both resveratrol and reference compounds were imported into AutoDock Vina via the PyRx 0.8 interface. Prior to docking, ligand structures underwent energy minimization through Open Babel using the Universal Force Field (UFF) and the conjugate gradient method for geometry optimization. The docking process involved targeting the protein’s active or binding pockets, which were defined using grid boxes encompassing key residues. Default docking parameters were applied throughout. Finally, the binding conformations were visually examined using Discovery Studio Visualizer version 16.

#### Binding assessment of resveratrol with α-synuclein

##### Preparation of protein crystal structure and receptor grid generation

The X-ray crystallographic structure of α-synuclein was obtained from the Protein Data Bank (https://www.rcsb.org/) using the PDB ID: 3Q25. Protein preparation was carried out using the Protein Preparation Wizard in the Schrödinger Glide Suite (version 2017–1), which corrected structural inconsistencies inherent in the crystallographic model. Hydrogen atoms that were missing were added, bond orders were assigned appropriately, and other parameters were maintained at default settings. Structural optimization was subsequently performed using the OPLS3 force field, with the root mean square deviation (RMSD) for heavy atoms constrained to 0.3 Å^91^.

A receptor grid was generated from the co-crystallized ligand (alpha maltose) within the binding pocket of 3Q25 using the Receptor Grid Generation tool in Schrödinger Suite 2017–1. This facilitated the automatic detection of the binding site coordinates (x = 4.77, y = 29.83, z = 7.53). Structural Data Files (SDF) for resveratrol and the reference ligand, levodopa (L-DOPA), were retrieved from PubChem (https://pubchem.ncbi.nlm.nih.gov) and imported into Maestro’s Schrödinger interface. For each compound, low-energy 3D conformers were generated with suitable bond lengths and angles. Ionization states were predicted at physiological pH (7.2 ± 0.2), and ligands were energy-minimized using the OPLS3 force field^113,114^. Docking of resveratrol and L-DOPA was performed using the Extra Precision (XP) mode of Glide, which provides more accurate and selective docking compared to Standard Precision (SP) and High Throughput Virtual Screening (HTVS) methods^115^. To validate the docking protocol, the original co-crystallized ligand was re-docked into the active site of 3Q25, and the results were used to evaluate docking accuracy.

Binding affinities of the docked complexes were further evaluated using Molecular Mechanics-Generalized Born Surface Area (MM/GBSA) calculations. This advanced computational approach, which integrates quantum mechanical corrections, was applied to reduce the likelihood of false positives from docking^95^. The docked ligands-3Q25 complexes were minimized by using the local optimization feature in Prime. The OPLS3 force field was employed to determine the binding energy (∆^bind^) for a set of ligands-3Q25 complexes. The Eq. (1) was used to calculate the binding free energy:

1 $$\Delta {\text{GBind}} = \Delta {\text{EMM}} + \Delta {\text{GSolv}} + \Delta {\text{GSA}}$$

where ΔEMM: is the variance between the minimized energy of the ligands- 3Q25 complexes, ΔGSolv: is the variation between the GBSA solvation energy of the ligands- 3Q25 complexes and the sum of the solvation energies for the protein and ligand.

#### Quantum chemical calculation

Resveratrol and the reference compound L-DOPA were subjected to structural optimization using Density Functional Theory (DFT) at the B3LYP/6-31G(d) level^116^ using Spartan 14 computational chemistry software. A restricted hybrid Hartree Fock-DFT self-consistent field calculation with Pulay’s direct inversion of the iterative sub-space and geometric direct minimization was employed^117^. The energies of the highest occupied molecular orbital (E_HOMO_), lowest unoccupied molecular orbital (E_LUMO_), and the energy band gap, E_g_ (Eq. 1) were calculated. The global reactivity descriptors were derived from the HOMO and LUMO energies (Eqs. 2–6).

2 $${\text{Eg }} = {\text{ E}}_{{{\text{LUMO}}}} - {\text{E}}_{{{\text{HOMO}}}}$$

3 $$\upeta \, = \frac{{E_{LUMO} - E_{HOMO} }}{2}$$

4 $$\updelta \, = \frac{1}{{\upeta }}$$

5 $$\upchi \, = \, - \frac{{E_{LUMO} + E_{HOMO} }}{2}$$

6 $${\text{C}}_{{\text{P}}} = \, -\upchi$$

#### Statistical analyses

Data were expressed as Mean ± Standard Deviation and statistical analyses were carried out with a one-way Analysis of Variance (ANOVA) followed by Tukey’s post hoc test using Graph Pad Prism (10). The survival rate curve was performed using the Kaplan–Meier survival plot and calculated by the log-rank (Mantel-Cox) test. Significant differences among the groups were determined at a 95% confidence interval, with *p* < 0.05 (n = 5 replicates) considered indicative of significance.

## Data Availability

All the experimental data have been provided within the manuscript. Any additional information can be requested from Amos Abolaji (ao.abolaji@ui.edu.ng).
